# INS-17 acts as a nutrient deprivation signal to mediate adult IIS-regulated associative behaviors in *C. elegans*

**DOI:** 10.1371/journal.pgen.1012130

**Published:** 2026-04-28

**Authors:** Emily J. Leptich, Priyadharshini Vijayakumar, Edward W. Pietryk, Meredith I. Williams, Rachana Rajupalem, Rachel N. Arey

**Affiliations:** 1 Department of Neuroscience, Baylor College of Medicine, Houston, Texas, United States of America; 2 Center for Precision Environmental Health, Baylor College of Medicine, Houston, Texas, United States of America; 3 Department of Molecular and Human Genetics, Baylor College of Medicine, Houston, Texas, United States of America; 4 Department of Molecular and Cellular Biology, Baylor College of Medicine, Houston, Texas, United States of America; University of California San Francisco, UNITED STATES OF AMERICA

## Abstract

Insulin/Insulin-like growth factor 1 (IGF-1) signaling (IIS) is a pleiotropic signaling pathway that functions across tissues to coordinate phenotypic changes in response to nutrient status. Thus, the ubiquity of the IIS pathway hinders efforts to elucidate the mechanisms driving specific IIS-related phenotypes. Previous research in the nematode worm *C. elegans* has demonstrated that loss of function of the IIS transmembrane receptor (IR) ortholog, DAF-2, results in a doubled lifespan and enhanced learning and memory behaviors in young and aged animals. However, these findings are the result of reducing DAF-2 receptor function rather than modulating ligand-receptor interactions. In the current study, we aimed to dissect ligand-receptor interactions that may regulate associative behaviors apart from canonical IIS lifespan phenotypes in *C. elegans*. To this end, we performed targeted genetic screening of Insulin-like Peptides (ILPs) previously identified as DAF-2 antagonists to test their role in learning and memory phenotypes. We discovered that only a single uncharacterized ILP, INS-17, is required for learning and memory. We also demonstrate that INS-17 is sufficient to confer extended memory ability and can promote the maintenance of learning and memory with age. Additionally, we observe that INS-17 regulates associative behaviors independent of lifespan, uncoupling some IIS-mutant phenotypes. We find that regulation of the *ins-17* genetic locus explains its unique requirement among ILPs for learning and memory behaviors. Finally, we found that INS-17 acts to signal a state of nutrient deprivation. This activity is required to properly process stimulus valence to promote advantageous behaviors. Our findings deepen the understanding of how IIS can regulate specific phenotypic outputs in response to changes in internal metabolic states.

## Author summary

Insulin signaling is a diverse actor that regulates many processes essential for life from growth and development, aging, cognition, among many others. However, how insulin signaling regulates myriad processes across tissues within an organism is poorly understood. Simple models, like the nematode worm *Caenorhabditis elegans*, highlight the diversity of insulin signaling function, where decreased insulin receptor function increases lifespan and enhances memory. Here, we use *C. elegans* to ask if specific receptor-ligand interactions regulate distinct insulin receptor-associated phenotypes. We found that one insulin-like peptide predicted to decrease insulin receptor function, INS-17, is uniquely required for learning and memory behaviors in young adult animals and that increasing INS-17 levels boosts memory performance in young and aged adults. Interestingly, these memory effects occur without any changes in lifespan, indicating that INS-17 may specifically regulate behavior in adulthood. Furthermore, we find that the regulation of the *ins-17* gene in the context of changing metabolic status is why it is important for behavior. Specifically, INS-17 acts as a signal that an animal is nutrient deprived and allows the organism to change its behavior accordingly. Our findings help to broaden our understanding of how insulin signaling integrates internal metabolic information to change phenotypic outputs.

## Introduction

The survival of most multicellular organisms depends on the coordination of complex physiological processes between multiple, functionally discrete tissues. Remarkably, each of these tissues carries out a diverse set of physiological functions using a highly overlapping set of cell regulatory mechanisms, including ubiquitous signaling pathways (e.g., MAPK/ERK, cAMP, and ubiquitin-proteasome pathway signaling [1–4]). Major components of these pathways are commonly pleiotropic, where a single gene can control multiple, distinct phenotypes depending on the context, such as life stage, cell type, and metabolic state. Across species, one prominent pleiotropic signaling pathway is the insulin/insulin-like growth factor 1 receptor signaling pathway, which regulates a wealth of phenotypes across multiple tissues, including growth and development, metabolism, fertility, aging, stress response, and survival [5–15]. Dissecting the specific signaling mechanisms by which IIS mediates such a wide range of phenotypic effects proves challenging, especially in mammalian systems. This is due to several factors, namely high costs to generate tools to examine pathways in a tissue-specific and temporally controlled manner; compensation between receptors, as a single tissue can regulate phenotypes organism-wide; and the time and resource burden to study certain IIS-regulated phenotypes, such as aging. However, invertebrate systems can help overcome these challenges, as IIS is highly conserved across species [9,10,14,16,17], and simple models allow for rapid, low-cost study of genes with remarkable temporal and tissue-specific precision [18–20].

The nematode *C. elegans* has been invaluable in elucidating the pleiotropy underlying IIS phenotypes. In fact, foundational experiments in the worm were the first to link insulin signaling and aging, where partial loss-of-function receptor mutants of the IR homolog, *daf-2*, have a doubled lifespan of around 40 days compared to the 20-day lifespan of wild-type animals [21,22]*.* Since this initial work, studies in IIS mutants revealed the pleiotropy of the DAF-2 receptor and its conserved downstream signaling components (*age-1*/PI3K and transcription factors *hsf-*1/HSF1, *skn-1*/NRF2, and *daf-16*/forkhead family (FOXO3)) in this relatively simple model [21–28], including that *daf-2* mutants have altered development, metabolism, fertility, motility, cognitive abilities, and more [12,26,29–39]. Collectively, research indicates that DAF*-2/*IR functions across tissues and is integral for sensing internal metabolic state along with environmental cues allowing for the worm to adapt appropriately to improve survival, though the importance of communicating metabolic state in all IIS-associated phenotypes remains unclear.

A major challenge in understanding IIS-dependent regulation of specific phenotypes is the ubiquity of the IIS pathway across tissues, where DAF-2/IR activity in some tissues can mask DAF-2/IR activity in other tissues. For example, it is well established that *daf-2* mutants have improved motility with age [30,40,41], but recent work using degron-based approaches found that reduction of *daf-2* function in neurons and muscles has opposing effects on maintenance of motility in aged animals [42]. This work sheds light on the remaining gap in our knowledge regarding how signaling through a single ubiquitous receptor can impact numerous phenotypes that are often reflective of the function of a relatively small number of cells. It is also unclear whether these distinct phenotypes can be uncoupled, and if so by what mechanisms.

Though the DAF-2 receptor and its downstream effectors have been well-studied, less is known about the upstream regulators of DAF-2/IR that drive specific phenotypic outputs. The *C. elegans* genome encodes 40 insulin-like peptides (ILPs) predicted to act as DAF-2 receptor agonists or antagonists, sometimes in a context-dependent fashion [43–47]. However, no single manipulation of an ILP fully reproduces reduced *daf-2* function phenotypes. ILPs also exhibit diverse and often non-overlapping expression patterns and are regulated by distinct physiological contexts [48]. Thus, we hypothesize that pleiotropic phenotypes exhibited by IIS mutants may also reflect distinct ligand-receptor interactions.

In the present study, we investigated if specific ILPs regulate *daf-2* mutant learning and memory phenotypes. We discovered that a single ILP, the DAF-2/IR antagonist INS-17, is required for learning and memory ability and is sufficient to promote memory. We also found that INS-17 is specifically required for IIS regulation of behavior and does not appear to be involved in other adult DAF-2 receptor-mediated phenotypes, including lifespan [47,49]. We find that the regulation of *ins-17* is the source of this phenotypic specificity. Finally, we investigated the significance of communicating changing metabolic status in associative behaviors and discovered that INS-17 acts as a nutrient-deprivation signal that regulates plastic behavior in the context of changing physiological states.

## Results

### INS-17 is specifically required for learning and memory ability

**Fig 1:**
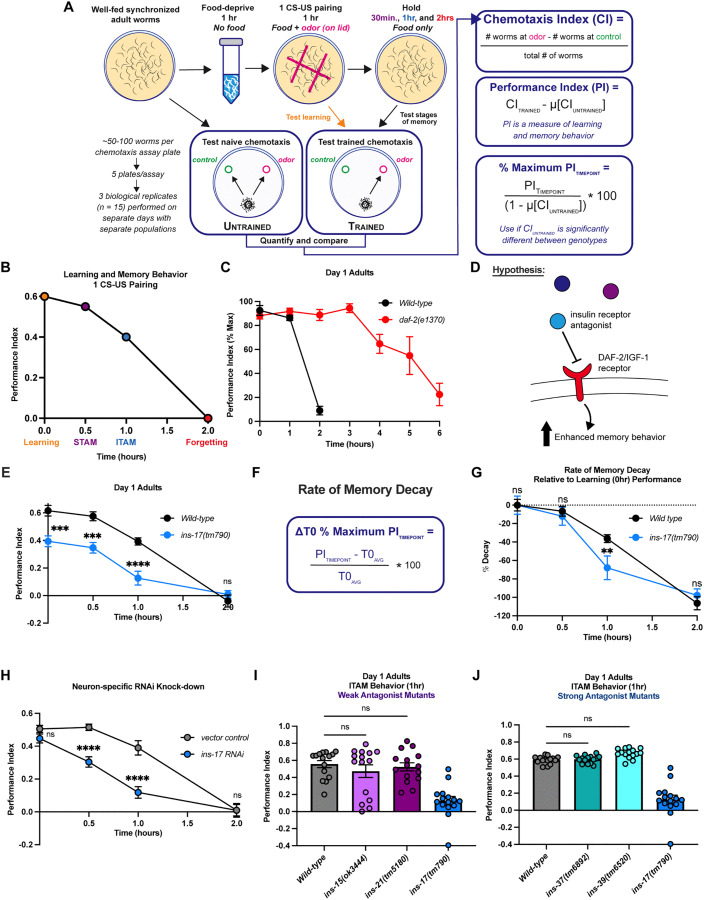
INS-17 is a novel and specific memory-regulating insulin receptor antagonist. **(A)** Diagram depicting positive associative olfactory learning and memory assay work-flow. Well-fed adult worms are food-deprived for 1 hour, conditioned for 1 hour with a food-odor (1 CS-US) pairing, and then either immediately tested for learning performance or moved to hold plates with food and tested for memory performance either 0.5 hours, 1 hour or 2 hours later. Memory performance is measured by subtracting the average untrained chemotaxis index from the trained chemotaxis index. % Maxmimum performance indices are used to compare performance indices between two genotypes that have significantly different baseline naïve chemotaxis. **(B)** Model graph of typical wild-type animal performance indices at learning, STAM, ITAM, and forgetting timepoints after undergoing the training paradigm. **(C)** Partial loss-of-function *daf-2(e1370)* mutants have extended STAM that lasts 6 hours compared to 2 hours for wild-type animals after 1 CS-US food-butanone pairing. % Max Performance reported as *daf-2(e1370)* animals have a higher baseline naïve attraction to neutral (10%) butanone concentration (see **S1A and S1B Fig**). Mean ± SEM. n = 10 per genotype. **(D)** Diagram depicting the hypothesis that insulin receptor antagonists may inhibit DAF-2 receptor function to promote learning and memory. **(E)** *ins-17* deletion mutants have impaired learning, STAM, and ITAM compared to wild-type worms. Two-way ANOVA with Bonferroni’s multiple comparisons test. Interaction between factors, p = 0.0002; timepoint, p < 0.0001; genotype, p < 0.0001. Mean ± SEM. n = 15 per genotype. ***p < 0.001, ****p < 0.0001; ns, not significant (p > 0.05). (**F)** Equation for calculating rate of memory decay relative to learning (T0, 0hr) performance. **(G)** *ins-17(tm790)* animals have a significantly higher rate of memory performance decay at the ITAM timepoint compared to wild-type animals. Two-way ANOVA with Bonferroni’s multiple comparisons test. Interaction between factors, p = 0.0901; timepoint, p < 0.0001; genotype, p = 0.2247. Mean ± SEM. n = 15 per genotype. **p < 0.01; ns, not significant (p > 0.05). **(H)** Adult-only, neuron-specific RNAi knock-down of *ins-17* leads to learning, STAM, and ITAM impairments compared to vector RNAi treated animals. Two-way ANOVA with Bonferroni’s multiple comparisons test. Interaction between factors, p = 0.0001; timepoint, p < 0.0001; RNAi treatment, p < 0.0001. Mean ± SEM. n = 15 per RNAi treatment. ****p < 0.0001; ns, not significant (p > 0.05). **(I)** Weak insulin receptor antagonist mutants for *ins-15* and *ins-21* and **(J)** strong insulin receptor antagonist mutants for *ins-37* and *ins-39* display no memory impairments. Depicted *ins-17* mutant behavior is ITAM performance from **(E)** and inserted for visual comparison purposes only, as experiments were performed on different days with separate populations. One-way ANOVA with Bonferroni’s multiple comparisons tests (p > 0.05, ns). Mean ± SEM. n = 15 per genotype. ns, not significant (p > 0.05).

Decreased DAF-2 receptor function results in a 3-fold extension in short- and intermediate associative memory (S/ITAM), as measured by positive olfactory association assays [31]. In this paradigm, animals form a positive association with the neutral odor butanone (10% in EtOH) when paired with food, which is measured by a training-dependent increase in butanone chemotaxis (Fig 1A and 1B [50]). A single food (unconditioned stimulus, US)-butanone (conditioned stimulus, CS) pairing normally results in molecularly conserved behaviors, including learning (0 hr), short-term memory (STAM, 0.5 hrs post-training), intermediate-term memory (ITAM, 1 hr post-training), and active forgetting over the course of 2 hours (Fig 1B). However, young adult *daf-2* mutants maintain a food-butanone association for up to 6 hours (Fig 1C [31]). Percent maximum (% Max) performance indices are shown for clarity (see Fig 1A for calculations), as *daf-2* animals have high naïve chemotaxis to 10% butanone in multiple studies (S1A Fig, [31,35,36,39]) that contribute to perceived learning impairments when graphed with raw performance indices (provided in S1B Fig). Given that partial loss-of-function *daf-2* animals display this significant extension in S/ITAM performance, we hypothesized that insulin receptor antagonist(s), which are predicted to reduce signaling through DAF-2/IR, may promote learning and memory behavior (Fig 1D). To test this hypothesis, we examined the learning and memory ability of publicly available DAF-2/IR antagonist mutants using this STAM paradigm. We tested learning and ITAM (1 hr post-training), as behavioral deficits are typically more prominent at this timepoint compared to STAM (0.5 hr post-training). Mutants tested included predicted strong (*ins -17, -37, and -39)* and weak (*ins-15, -21,* and *-22)* antagonists [47,51,52]. However, we recently determined that mutants for *ins-22* have an abnormally high preference for 10% butanone (S1L Fig, [53]). Thus, we excluded *ins-22* from our targeted behavioral screen, as this high naïve chemotaxis would make it difficult to measure training-dependent increases in butanone preference. Interestingly, only loss-of-function mutants of *ins-17* exhibited behavioral deficits: these include significant deficits in learning, as well as lower STAM and ITAM performance (Fig 1E). We sought to determine if the altered memory behaviors we observed were indeed deficits, and not just reflective of poor learning. We therefore calculated the rate of memory decay for both wild-type and *ins-17* mutants, which takes into account the decrease in memory performance of a given genotype relative to the initial learning performance (for calculations see Fig 1F). We found *ins-17* mutants showed a more significant rate of decay than wild-type animals specifically at the ITAM timepoint (Fig 1G), suggesting that *ins-17* likely is required for memory as well. These deficits were not accompanied by impaired sensory function, as measured by naïve chemotaxis assays to the highly attractive concentration (0.1%) of butanone (S1C-S1D Fig), suggesting that they are specific to learning and memory. INS-17 is a relatively uncharacterized ILP; aside from its prediction as a strong antagonist, previous research has established *ins-17’*s role in regulating dauer formation in early development [49]. To our knowledge, INS-17 is uncharacterized regarding complex behavior, and these results (Fig 1E-1G) suggest that it is a novel learning and memory-regulating ILP. Strikingly, other antagonist mutants behaved in a manner that was indistinguishable from wild-type animals, including baseline naïve chemotaxis (Figs 1I-1J and S1H-S1K).

Finally, we sought to both bypass potential behavioral deficits from loss of *ins-17* during development, and to identify the tissue where INS-17 is required to regulate associative behavior. Since INS-17 is highly expressed in the nervous-system, we hypothesized that neurons may use INS-17 as a memory signal. Using a neuron-specific RNAi-sensitive line [55], we performed an adult-only *ins-17* knockdown. We found that neuron-specific loss of *ins-*17 only in adulthood did not affect learning ability, but observed significant S/ITAM behavioral deficits in *ins-17 RNAi* knock-down animals (Fig 1H, for memory decay S1G Fig)—similar to what we observed in deletion mutants (Fig 1E)—with no detectable effects on sensory ability (S1E-S1F Fig). This suggests that the learning deficits observed in the *ins-17* mutants may be developmental in origin and demonstrates that INS-17 is likely released from neurons to regulate associative behaviors.

### INS-17 signals through DAF-2 and promotes memory

**Fig 2:**
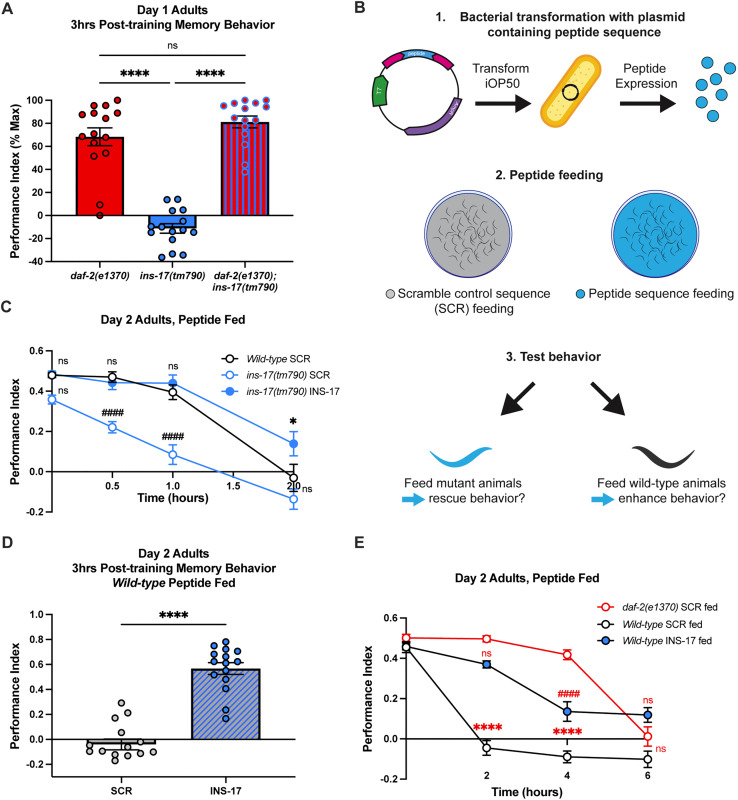
INS-17 and DAF-2 are epistatic and promote memory. **(A)** *daf-2* and *ins-17* share an epistatic relationship. 3hrs post-training (1 CS-US pairing), *daf-2(e1370)* mutants and *daf-2(e1370)*;*ins-17(tm790)* double mutants display memory behavior while *ins-17(tm790)* animals display no memory behavior. One-way ANOVA with Bonferroni’s multiple comparisons tests (p < 0.0001). Mean ± SEM. n = 15 per genotype. ****p < 0.0001; ns, not significant (p > 0.05). **(B)** Diagram of microbial feeding paradigm. iOP50 bacteria were transformed with a plasmid containing either the peptide sequence of interest or a scramble control sequence and then fed to worms before testing behavioral effects. **(C)** INS-17 peptide feeding rescues *ins-17(tm790)* learning, STAM and ITAM behaviors, while SCR fed mutants display significant S/ITAM impairments compared to SCR fed wild-type animals. Two-way ANOVA with Bonferroni’s multiple comparisons tests. Interaction between factors, p = 0.0157; timepoint, p < 0.0001; genotype/treatment; p < 0.0001. Mean ± SEM. n = 15 per feeding treatment.* and # is significance relative to SCR fed wild-type animal performance. *p < 0.05. ###p < 0.001, ####p < 0.0001; ns, not significant (p > 0.05). **(D)** Wild-type animals fed INS-17 peptides display 3-hour memory behavior after 1 CS-US pairing. Mann-Whitney test comparing ranks. Mean ± SEM. n = 15 per peptide treatment. ****p < 0.0001. **(E)** Wild-type animals fed INS-17 displayed memory behavior 2 hours post-training not significantly different from *daf-2(e1370)* animals fed SCR, while SCR fed wild-type animals displayed forgetting behavior at this timepoint. Four hours after training, INS-17 fed wild-type animals showed significantly lower memory performance compared to SCR fed *daf-2(e1370)* animals, which remembered up until 6 hours after training. Two-way ANOVA with Bonferroni’s multiple comparisons tests. Mean ± SEM. n = 15 per feeding treatment. Interaction between factors, p < 0.0001; timepoint, p < 0.0001; genotype/treatment, p < 0.0001. * and # is significance relative to SCR fed *daf-2(e1370)* performance. ****p < 0.0001; ns, not significant (p > 0.05).

We next wanted to confirm that INS-17 signaling was indeed mediated by the DAF-2 receptor and tested the learning and memory behavior of *daf-2(e1370);ins-17(tm790)* double mutants. We examined double mutant memory behavior at 3 hours after 1 CS-US pairing, a timepoint where *daf-2* mutants exhibit extended memory [31]. *daf-2(e1370);ins-17(tm790)* worms demonstrated extended 3-hour memory indistinguishable from *daf-2* animals (Figs 2A and S2A-S2C). Thus, we infer from these results that DAF-2/IR is epistatic to INS-17 in the regulation of learning and memory.

Next, we wanted to explore how manipulating levels of INS-17 may influence behavioral outputs. Our behavior paradigm tests an adult-specific behavior [39], and *ins-17* regulates dauer in development [49], so we sought to avoid confounding our results with developmental effects of traditional transgenic approaches. Recently, we developed a novel microbial feeding paradigm to manipulate neuropeptide signaling specifically in adult animals [53]. In these experiments, *C. elegans* are fed *E. coli* bacteria expressing a plasmid containing the sequence encoding a neuropeptide of interest flanked by processing enzyme cut sites (Fig 2B). Like feeding-based RNAi approaches [54], this method offers precise temporal control of increasing neuropeptide levels via adult-only manipulation to avoid potential effects on early development. We have demonstrated that this feeding method could work in an ‘overexpression’ manner when administered to wild-type animals, altering sensory behavior [53]. Here, we wanted to extend this approach to assess if administering additional INS-17 to adult animals could affect associative behaviors. We hypothesized that increasing the levels of a memory-regulating *daf-2* antagonist would result in enhanced memory ability.

To first establish the ability of this method to effectively manipulate INS-17 signaling, we tested if INS-17 peptide feeding was sufficient to rescue learning and memory behavior of *ins-17(tm790)* animals. Briefly, we fed L4 animals *E. coli* expressing either INS-17 or a scrambled control peptide (SCR) and examined their behavior at Day 2 of adulthood. SCR fed mutants still exhibited behavioral deficits, while adult-only feeding of INS-17 to *ins-17* deficient mutants was sufficient to rescue their behavior, which resembled the behavior of wild-type animals fed SCR peptide (Fig 2C). Notably, we determined these findings were not the result of altered sensory function in these animals (S2D-S2E Fig). We next explored if INS-17 peptide feeding was sufficient to boost wild-type animal memory behavior past the 2-hour timepoint after 1 CS-US pairing. Remarkably, we found that wild-type animals fed INS-17 during adulthood displayed 3-hour memory post-training, while SCR fed animals demonstrated no memory phenotype, as expected (Fig 2D). We confirmed that peptide feeding to both wild-type and mutant animals did not alter baseline chemosensation, indicating the effect is memory-specific (S2F-S2G Fig). These results suggest that increasing INS-17 is sufficient to boost cognitive performance and phenocopy *daf-2* animals’ 3-hour memory behavior.

We next asked if the improvements we saw in behavioral performance after supplementation with INS-17 could also extend to learning. Previous work found that animals need at least a 30 minute food-butanone pairing in order exhibit a positive butanone association (learn, [31]). We tested the effects of INS-17 supplementation after sub-threshold conditioning (15 minute food-butanone pairing). We found, in agreement with previous results, that SCR-fed control animals failed to learn after this short pairing (S2H Fig) and INS-17 had no detectable effect on performance, suggesting that supplementation specifically promotes memory (S2H Fig). We also confirmed that the memory-extending effects of INS-17 supplementation were due specifically to interactions with DAF-2/IR. We fed INS-17 to *daf-2* mutants and saw no marked improvement in memory-extension (S2I-S2J Fig), suggesting that INS-17 enhances memory through the DAF-2 insulin receptor.

We next examined if supplementation of INS-17 was sufficient to extend memory in wild-type animals for the full 6 hours observed in *daf-2* mutants. Intriguingly, wild-type animals supplemented with INS-17 showed poorer performance than *daf-2* mutants beyond 4 hours post-training (Fig 2E, for naïve butanone preferences see S2I Fig). Recently, the memory extension observed in *daf-2* mutants was found to require the activity of two different tissues – memory up to 3 hours post-training required DAF-2/IR in the nervous system, while 4–6 hour post-training memory is mediated by DAF-2/IR in the hypodermis [36]. This suggests that INS-17 may specifically interact with DAF-2/IR in neurons to promote memory.

### INS-17 partially regulates the cognitive aging phenotypes mediated by the DAF-2 receptor independent of lifespan

**Fig 3:**
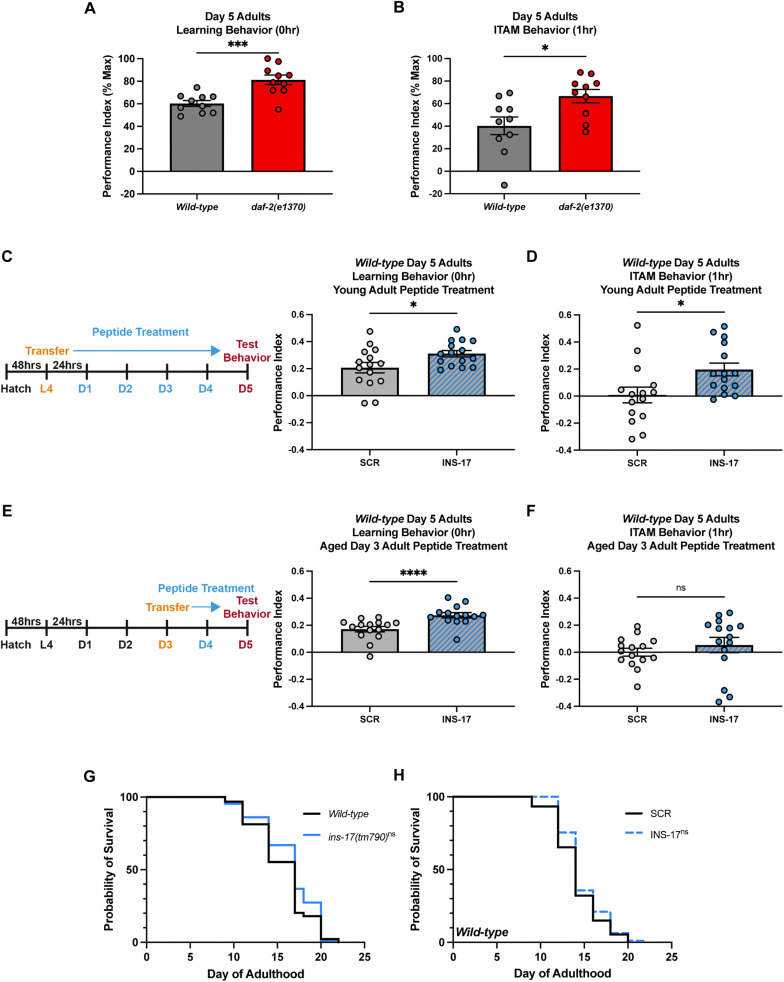
INS-17 regulates cognitive aging phenotypes observed in *daf-2* mutants. **(A)** Day 5 adult *daf-2(e1370)* mutants display better learning and **(B)** memory behavior compared to wild-type worms. Mann-Whitney tests to compare ranks. Mean ± SEM. n = 10 per genotype. *p < 0.05, *** p < 0.001. **(C)** Timeline of young adult peptide treatment. Wild-type L4 animals are fed SCR or INS-17 iOP50 bacteria until Day 5 of adulthood when S/ITAM behavior is tested. INS-17 peptide treated worms have increased learning and **(D)** ITAM performance compared to SCR peptide treated animals. Mann-Whitney tests to compare ranks. Mean ± SEM. n = 15 per peptide treatment. *p < 0.05. **(E)** Timeline of aged adult peptide treatment. Wild-type Day 3 adult animals are fed SCR or INS-17 iOP50 bacteria until Day 5 of adulthood when S/ITAM behavior is tested. INS-17 peptide treated worms have increased learning performance, but not **(F)** ITAM performance, compared to SCR peptide treated animals. Mann-Whitney tests to compare ranks. Mean ± SEM. n = 15 per peptide treatment. ****p < 0.0001; ns, not significant (p > 0.05). **(G)** Probability of survival analysis demonstrates there is no significant difference in lifespan comparing wild-type animals and *ins-17(tm790)* animals. One replicate depicted is representative of three replicates (see S3F **and** S3G Fig). Mean/median ± SEM. n ≥ 100 per replicate. ns, not significant (p > 0.05). **(H)** Probability of survival analysis for wild-type animals treated with SCR peptide compared to wild-type animals fed INS-17 peptide shows no significant difference in average lifespan. One replicate depicted is representative of three replicates (see S3H **and** S3I Fig). Mean/median ± SEM. n ≥ 100 per replicate. ns, not significant (p > 0.05).

In addition to exhibiting extended STAM at young adult stages, *daf-2* mutants also maintain the ability to learn and remember after 1 CS-US pairing with age [31]. Wild-type animals display age-related cognitive deficits as early as Days 3 and 5 of adulthood (Figs 3A-3B and S3A-S3C), while *daf-2* mutants maintain performance akin to Day 1 of adulthood at these timepoints. Therefore, we wanted to ask if INS-17 could also be involved in these age-related phenotypes associated with altered *daf-2* function. As *ins-17* mutants already display severe behavioral defects at Day 1 of adulthood, we examined the effect of increased INS-17 on age-related behavioral impairments in wild-type animals, again using a peptide feeding-based approach [53].

We first assessed the behavior of animals supplemented continuously with INS-17 from the L4 stage until Day 5 of adulthood (Fig 3C), when worms are considered cognitively aged [31]. INS-17 fed animals demonstrated significantly better learning and ITAM performance compared to wild-type SCR fed controls (Figs 3C-3D and S3D). Next, we wondered if manipulating the DAF-2/IR pathway via INS-17 later in life could influence cognitive aging, as previous work has shown that degradation of DAF-2/IR via an auxin inducible degron system in older animals results in improved STAM [36]. We subjected Day 3 adult wild-type animals (which already display age-related cognitive deficits compared to Day 1 Adults (Fig 3A)) to SCR or INS-17 peptide feeding and tested their learning and memory abilities at Day 5 of adulthood (Fig 3E). Notably, we found that Day 5 INS-17 fed animals had better learning behaviors compared to SCR fed controls (Figs 3E-3F and S3E), while ITAM was unaffected. These findings suggest that INS-17 is a DAF-2/IR ligand that could partially contribute to the enhanced cognitive ability with age observed when DAF-2/IR activity levels are reduced. The inability of INS-17 supplementation to fully recapitulate *daf-2* cognitive aging phenotypes is likely reflective of our findings that INS-17 may preferentially interact with neuronal DAF-2 (Fig 2E), which contributes significantly less to cognitive aging phenotypes than hypodermal DAF-2 [36].

Since *daf-2* animals have both a doubled lifespan and enhanced memory phenotypes in young and aged animals [21,31], we wanted to determine if manipulating INS-17*,* as a strong DAF-2/IR antagonist, would alter lifespan in addition to associative behaviors. As previously reported in the literature [49], we confirmed that *ins-17(tm790)* animals have no significant difference in lifespan to wild-type animals (Figs 3G and S3F-S3G). We also tested the lifespans of wild-type animals fed SCR compared to those fed INS-17 in adulthood, finding no significant differences between groups (Figs 3H and S3H-S3I). Thus, our findings demonstrate that either loss of *ins-17* or increased levels of INS-17 has no effect on adult lifespan, highlighting that INS-17 does not likely regulate all adult DAF-2 receptor-mediated phenotypes.

### INS-17 engages multiple pathways downstream of DAF-2 to regulate learning and memory

**Fig 4:**
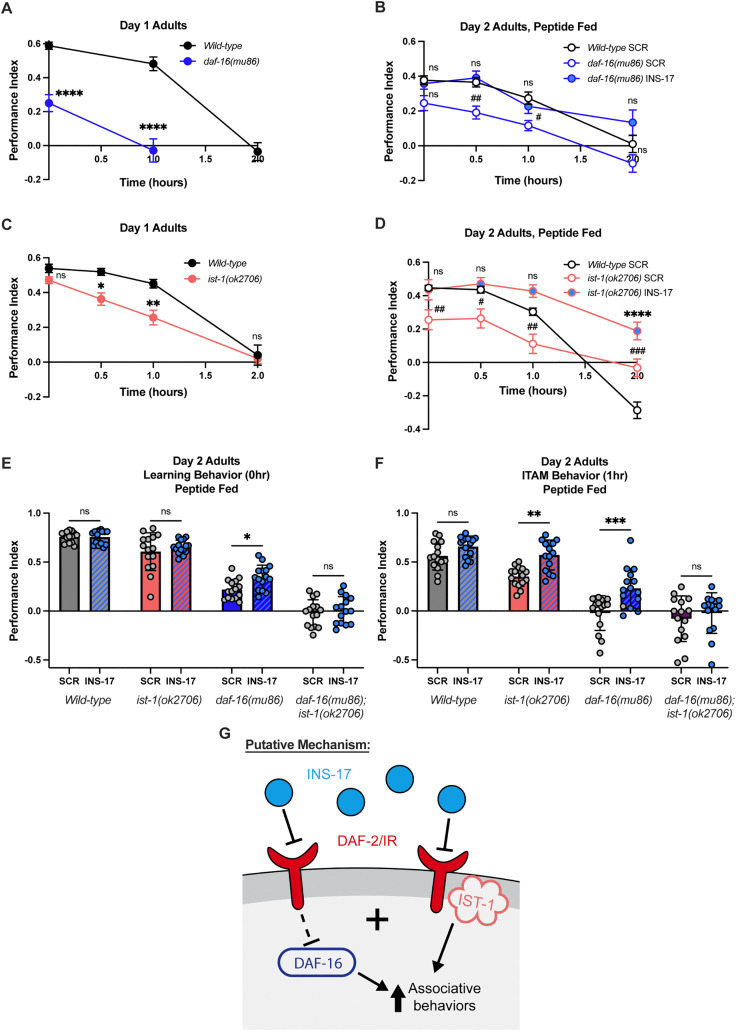
INS-17 likely engages parallel pathways of DAF-2 receptor signaling. **(A)** *daf-16(mu86)* deletion mutants have impaired learning and memory behavior compared to wild-type animals. Two-way ANOVA with Bonferroni’s multiple comparisons tests. Interaction between factors, p < 0.0001; timepoint, p < 0.0001; genotype, p < 0.0001. Mean ± SEM. n = 10 per genotype. ****p < 0.0001. **(B)** INS-17 peptide treatment rescues *daf-16(mu86)* S/ITAM impairments. Two-way ANOVA with Bonferroni’s multiple comparisons tests. Interaction between factors, p = 0.4342; timepoint, p < 0.0001; genotype/treatment, p < 0.0001. Mean ± SEM. n = 15 per genotype and peptide treatment. Wild-type animals fed INS-17 performance indices are in S4C Fig. # is significance relative to wild-type. #p < 0.05, ##p < 0.01; ns, not significant (p > 0.05). **(C)** *ist-1(ok2706)* deletion mutants display a slight but not significant learning deficit and impaired S/ITAM behaviors compared to wild-type worms. Two-way ANOVA with Bonferroni’s multiple comparisons tests. Interaction between factors, p = 0.0446; timepoint, p < 0.0001; genotype, p < 0.0001. Mean ± SEM. n = 15 per genotype. *p < 0.05, **p < 0.01; ns, not significant (p > 0.05). **(D)** INS-17 peptide treatment rescues *ist-1(ok2706)* learning and memory impairments. Two-way ANOVA with Bonferroni’s multiple comparisons tests. Interaction between factors, p < 0.0001; timepoint, p < 0.0001; genotype, p < 0.0001. Mean ± SEM. n = 15 per genotype and peptide treatment. * and # is significance relative to wild-type. #p < 0.05, ##p < 0.01, ###p < 0.001, ****p < 0.0001; ns, not significant (p > 0.05). **(E)** *daf-16(mu86)*;*ist-1(ok2706)* animals display significant learning and **(F)** memory impairments that are not rescued by INS-17 peptide feeding, while *daf-16(mu86)* and *ist-1(ok2706)* deficits are rescued by INS-17 peptide administration. Two-way ANOVA with Bonferroni’s multiple comparisons tests. For **4E**, interaction between factors, p = 0.2161; treatment, p < 0.0227; genotype, p < 0.0001. For 4F, interaction between factors, p = 0.0816; treatment, p < 0.0001; genotype, p < 0.0001. Mean ± SEM. n = 14-15 per genotype and peptide treatment *p < 0.05, **p < 0.01, ***p < 0.001; ns, not significant (p > 0.05). **(G)** Proposed mechanism for DAF-16 and IST-1 signaling down-stream of DAF-2/IR that may both be regulated by INS-17 peptide release to promote associative behaviors.

We next sought to identify the pathways downstream of DAF-2/IR that could explain the phenotypic specificity of INS-17. The canonical downstream target of DAF-2/IR signaling is the FOXO transcription factor homolog DAF-16 [27,28], which is required for the doubled lifespan extension of *daf-2* loss-of-function worms [26,28,55]. Additionally, *daf-16* mutants exhibit severely impaired learning and memory phenotypes (Figs 4A and S4A [31]). Since *ins-17* is required for positive associative memory behavior and there are no adult lifespan phenotypes associated with *ins-17* (Fig 3G-3H), we hypothesized that *ins-17* activates a pathway that acts in parallel to *daf-16*. To test this, we examined if INS-17 feeding could improve the memory impairments of *daf-16* worms. We found that adult-only INS-17 feeding rescued the learning and memory of *daf-16(mu86)* animals to SCR fed wild-type performance levels, while SCR fed *daf-16(mu86)* worms still displayed behavioral deficits (Figs 4B and S4B-S4C). These results support that INS-17 could act independently of DAF-16 to regulate associative behaviors.

Interestingly, recent research has uncovered evidence of pathways that can regulate cognitive abilities downstream of DAF-2/IR in parallel to DAF-16. This includes the discovery of a novel role for the IR substrate IST-1, which has been shown to function partially in parallel to DAF-16 to regulate learning ability in an aversive olfactory training paradigm [56]. Thus, we aimed to determine if *ins-17* is functioning in the same pathway as *ist-1*. First, we examined if *ist-1* is required in our positive associative memory paradigm. We found that *ist-1(ok2706)* animals have impaired positive associative STAM behavior compared to wild-type animals (Fig 4C). Like *daf-16* animals, we performed adult-only INS-17 feeding with *ist-1(ok2706)* worms and found that INS-17 peptide feeding was sufficient to boost *ist-1* mutant S/ITAM performance to a level similar to wild-type animals fed SCR, while *ist-1* animals fed SCR still displayed deficits (Fig 4D). Furthermore, we confirmed *ist-1* deficits were not the result of altered baseline chemosensation of butanone nor peptide feeding (S4D-S4G Fig).

While IST-1 and DAF-16 can function in parallel, it is possible that these two pathways can compensate for one another. Indeed, Cheng et al., (2022) identified that while *ist-1* could regulate learning independently of *daf-16*, *daf-16* was still required for this behavior [56]. We hypothesized that INS-17 could similarly engage both *daf-16* and *ist-1* to promote memory ability, and that partial regulation of either arm of the DAF-2 receptor signaling pathway could act as a compensatory mechanism in the mutant backgrounds when fed INS-17. Therefore, we examined if the beneficial effects of INS-17 administration were abolished when both genes were mutated. We generated *daf-16(mu86);ist-1(ok2706)* double mutants and observed that they had significant learning and memory deficits (Fig 4E-4F) compared to either single mutant alone. Because of the striking nature of these deficits, we confirmed that double mutant animals still maintained chemosensory ability and determined that they exhibited preference for chemoattractants (S4H-S4I Fig). We found that INS-17 peptide administration failed to rescue these severe behavioral defects, in contrast to the improvement observed in single mutants (Figs 4E-4F, S4J-S4K). These results suggest that INS-17 feeding can activate DAF-16 and IST-1, partially in parallel, to regulate associative behaviors (for schematic of proposed pathway, see Fig 4G).

### Regulation of the *ins-17* genetic locus is required for proper regulation of behavior

While we had identified how INS-17 might regulate distinct DAF-2/IR mediated phenotypes, we also sought to examine what made INS-17 unique relative to other antagonists in regard to regulating behavior. Interestingly, though the *C. elegans* genome encodes 40 ILPs, there are only three structurally similar antagonists (*ins-15, -17, and -37*) known as γ-insulins, which have three disulfide bonds similar to vertebrates; these genes are encoded across multiple genomic regions (ch. II and III) and thus likely under the control of distinct regulatory elements [43,47,57]. We therefore hypothesized that phenotypic specificity was not necessarily due to insulin class or structure, but instead via *ins-17*’s regulation by its surrounding genomic elements, which likely respond to different signaling pathways and environmental cues. To test this hypothesis, we explored if replacing *ins-17* with another strong DAF-2/IR antagonist was sufficient to maintain normal positive associative memory ability in an *ins-17* mutant background.

**Fig 5:**
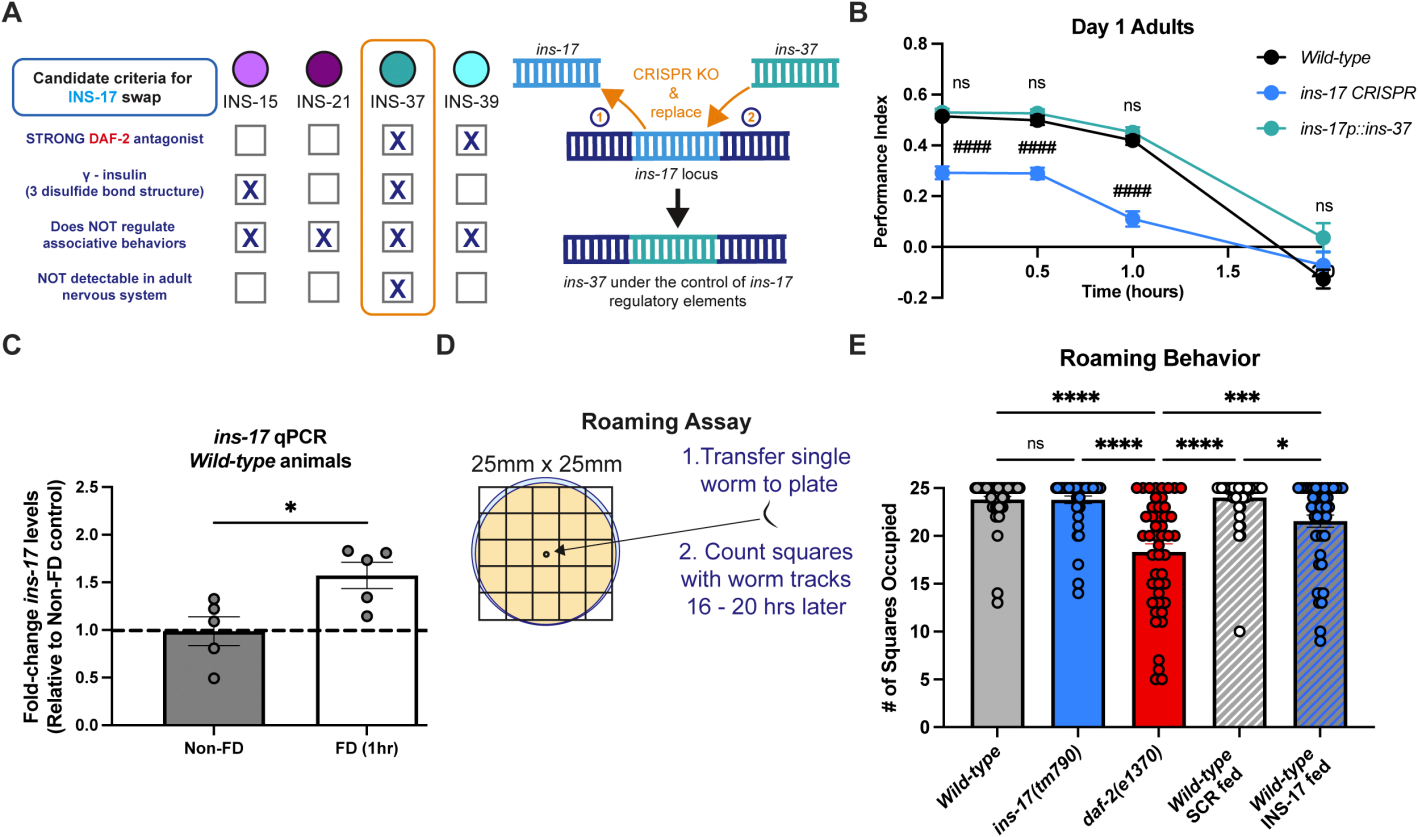
*ins-17* rapidly responds to nutrient deprivation and communicates metabolic status. **(A)** Schematic depicting criteria met by each insulin receptor antagonist peptide to qualify for the ‘insulin swap’ experiment (left) as well as a diagram showing the *ins-17* replacement experiment design with *ins-37* (right). *ins-17* was removed with CRISPR and replaced with *ins-37* at the *ins-17* locus so *ins-37* would be expressed at endogenous *ins-17* levels. **(B)** Replacing *ins-17* with *ins-37* has no significant effect on behavior compared to wild-type animals, while *ins-17* CRISPR KO (*ins-17(knu1320)*) animals have impaired learning and S/ITAM. Two-way ANOVA with Bonferroni’s multiple comparisons tests. Interaction between factors, p < 0.0001; timepoint, p < 0.0001; genotype, p < 0.0001. Mean ± SEM. n = 15 per genotype. # is significance relative to wild-type. ####p < 0.0001; ns, not significant (p > 0.05). **(C)** qRT-PCR of *ins-17* mRNA levels in Day 1 adult wild-type animals show that one hour of food-deprivation (FD) results in increased *ins-17* transcript levels. The dotted line represents a fold-change of 1 relative to the average of non-FD control values, indicating no change between conditions at that level. Mann-Whitney test to compare ranks. Mean ± SEM. n = 5 plates, ~ 1000 worms each, per genotype. *p < 0.05. For levels measured after two hours of food-deprivation, please see S5C Fig**. (D)** Schematic depicting the roaming assay work-flow, where a single L4 animal is transferred to a 35mm petri dish with solid media and food and allowed to roam for 16–20 hours; subsequently, worm tracks are counted by superimposing a 25mm x 25mm grid underneath the plate and recording the number of squares that overlap with the tracks. **(E)** Roaming behavior of wild-type, *ins-17(tm790)*, and *daf-2(e1370)* animals, as well as wild-type animals fed either SCR or INS-17 peptides. One-way ANOVA with Bonferroni’s multiple comparisons tests (p < 0.0001). Mean ± SEM. n = 20–25 worms per genotype or treatment. *p < 0.05, ***p < 0.001, ****p < 0.0001; ns, not significant (p > 0.05).

We selected *ins-37* to swap in for *ins-17* due to a key overlap in structure, as both ILPs are predicted strong DAF-2/IR antagonists and are γ-insulins, which are defined by three di-sulfide bonds [47]. The other antagonistic γ-insulin, *ins-15,* is a predicted weak antagonist, making it a less attractive candidate, while the other strong antagonist, *ins-39*, is an α-insulin, and contains additional intra-chain disulfide bonds making it structurally dissimilar. Moreover, *ins-37* mutants had no observable learning and memory impairments (Figs 1J and S1K) and *ins-37* is the only antagonistic γ-insulin not detectable in adult neurons [39,58,59], making it an ideal strong antagonist to test our hypothesis (For schematic of candidate selection, see Fig 5A).

Thus, we generated an *ins-17* CRISPR knockout to serve as a platform for our “insulin swap” experiment (Fig 5A). Subsequently, we examined if a CRISPR-replacement of *ins-37* at the *ins-17* locus in these CRISPR-knockout animals, which should lead to it being expressed at endogenous *ins-17* levels, is sufficient for normal learning and memory behavior. First, we confirmed that the newly generated *ins-17* CRISPR knockout line exhibited learning and memory impairments, and saw that they had behavioral deficits similar to the predicted loss-of-function mutants (Figs 1C, 5B and S5A-S5B). Interestingly, we found that replacing *ins-17* with *ins-37* could rescue the defective learning and memory of *ins-17* KO animals, functionally replacing endogenous *ins-17* in our assay (Figs 5B and S5A-S5B). These results suggest that the genomic context in which *ins-17* is regulated underlies its importance in promoting specific behavioral phenotypes.

### *ins-17* expression rapidly responds to nutrient deprivation and communicates internal metabolic status

Next, we wanted to uncover the physiological conditions that regulate *ins-17* expression and assess their role in learning and memory. We mined publicly available gene expression datasets and discovered that *ins-17* transcription is likely regulated by metabolic state, as it is consistently upregulated in multiple starvation paradigms and genetic models of nutrient deprivation (S1 Table) [39,49,51,60–62]. Interestingly, our appetitive behavioral paradigm involves a 1-hour food deprivation (FD) step prior to the 1 CS-US (food-butanone) pairing, which has been reported to promote a more robust CS-US association compared to animals that do not undergo a FD period prior to memory training [31,63]. We first examined if the FD step incorporated into our paradigm was sufficient to increase *ins-1*7. We performed qPCR analysis to measure *ins-17* levels in wild-type worms after 1 hour of FD and found that even this short time period was sufficient to observe a significant increase in *ins-17* expression (Fig 5C). We also observed potentially higher levels of *ins-17* following 2 hours of FD (S5C Fig), suggesting that this locus rapidly responds to changes in nutrient availability. We next asked if conditioning itself would affect *ins-17* expression. We found that *ins-17* levels were not significantly different between conditioned and naïve animals (S5D Fig). These findings are in agreement with previous work that no new transcription is required during conditioning for learning and memory after 1 CS-US pairing [64], and that *ins-17* levels drop upon re-feeding after food deprivation [51,62].

We then hypothesized that these rapid changes in *ins-17* expression in response to even brief food deprivation allowed it to serve as a signal of internal metabolic status. *C. elegans* exhibits two-distinct behavioral states in response to changing internal metabolic state, roaming and dwelling [65–70], where animals tend to dwell more in a starved state or in mutants that mimic nutrient deprivation, including *daf-2* mutants [70]. These behaviors can be easily quantified by measuring exploration behavior across a lawn of OP50 *E. coli* on a small plate marked with a grid (for schematic, see Fig 5D), and counting the number of squares occupied by an individual worm over 16–20 hours [67]. We compared the roaming behavior of wild-type animals, *ins-17* and *daf-2* mutants, and SCR or INS-17 supplemented wild-type animals. We found, as previously described [70], *daf-2* mutants showed less roaming (more dwelling, Fig 5E), consistent with the idea that they sense they are in a nutrient-deprived state. Interestingly, *ins-17* mutants did not differ significantly from wild-type animals in roaming behavior; however, INS-17 supplementation during adulthood reduced the roaming behavior of wild-type animals (Fig 5E). This indicates that INS-17 acts as a signal to communicate internal metabolic status, specifically nutrient deprivation, to the nervous system.

### INS-17 is required for properly sensing internal nutrient status to allow for processing of stimulus valence

**Fig 6:**
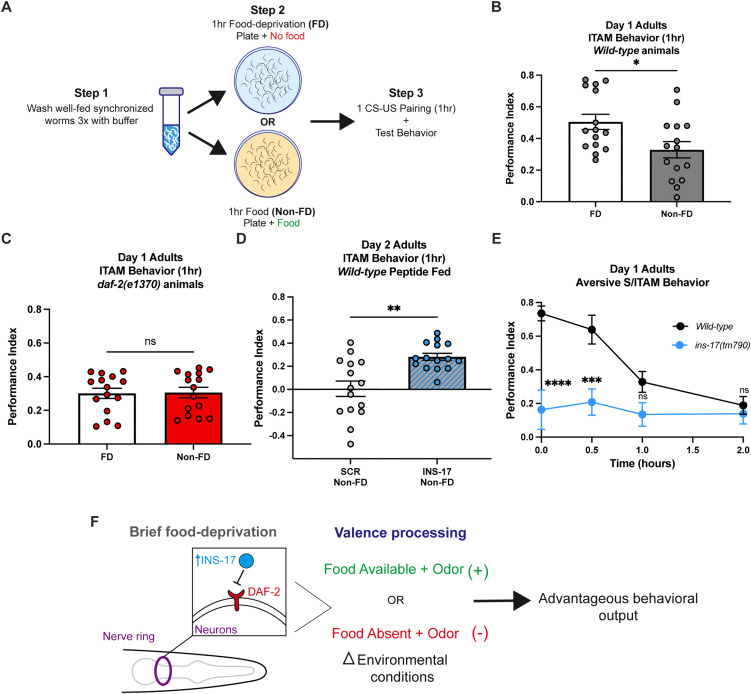
INS-17 is regulated by nutrient deprivation to promote advantageous behaviors. **(A)** Schematic depicting food-deprivation experiment workflow: well-fed adult worms are washed three times in M9 buffer solution to remove bacteria and placed on 100mm agar plates either seeded with a bacterial lawn (non-FD) or no lawn (FD) prior to training and behavior testing. **(B)** Wild-type animals display more robust ITAM behavior if a FD step is administered prior to 1 CS-US pairing compared to training alone (non-FD conditions). Mann-Whitney test to compare ranks. Mean ± SEM. n = 15 per condition. *p < 0.05. **(C)** Food-deprivation prior to training (1 CS-US pairing) does not enhance *daf-2(e1370)* ITAM performance. Mann-Whitney test to compare ranks. Mean ± SEM. n = 14-15 per condition. ns, not significant (p > 0.05). **(D)** INS-17 peptide treatment in wild-type animals is sufficient to promote robust ITAM performance in non-FD conditions prior to training compared to non-FD SCR fed animals. See S6F Fig for food-deprived SCR and INS-17 peptide fed comparisons. Mann-Whitney test to compare ranks. Mean ± SEM. n = 15 per peptide treatment. **p < 0.01. **(E)** *ins-17(tm790)* mutants have significantly impaired aversive associative learning performance compared to wild-type worms. Absolute values of learning and memory performance indices are reported. Two-way ANOVA with Bonferroni’s multiple comparisons tests. Interaction between factors, p = 0.0034; timepoint, p = 0.0003; genotype, p < 0.0001. Mean ± SEM. n = 10 per genotype. ***p < 0.001, ****p < 0.0001; ns, not significant (p > 0.05). **(F)** Putative diagram of how INS-17 is upregulated in the nervous system in response to brief food-deprivation to signal the valence of positive and aversive changes in environmental conditions to inform behavioral outputs advantageous for survival.

We next asked the significance of sensing internal metabolic status to associative behaviors. Based on our gene expression and roaming behavior results, we hypothesized that INS-17 could promote memory performance by functioning as a FD signal. We therefore assessed the role of insulin signaling in FD-associated improvements in associative behavior. We first confirmed that FD could indeed improve memory ability by comparing the behavioral performance of wild-type animals that underwent FD prior to training to animals that were not food-deprived (non-FD) prior to training (Fig 6A). Similar to previous reports [31], we found that wild-type FD animals have significantly better ITAM performance, which is measured one hour post-training, compared to non-FD animals (Figs 6B, S6A and S6B). Interestingly, *daf-2* mutant phenotypes are strongly associated with phenotypes paralleling those of chronically starved worms [66,71–73], so we hypothesized that *daf-2* animals may not require the FD step prior to 1 CS-US pairing for robust ITAM behavior, as they are thought to have a perceived nutrient status of chronic starvation even when well-fed. We found that non-FD *daf-2* animals that underwent 1 CS-US pairing had ITAM behavior nearly identical to *daf-2* animals that were FD prior to training (Figs 6C and S6C), indicating that they might not respond to the memory promoting effects of FD, and may not require FD for robust ITAM. Altogether, these results indicate that *daf-2* animals’ improved memory phenotype could be driven by their sensation of a chronically starved internal state.

We next tested if INS-17’s function as a FD signal was important for associative behavior. If this was the case, we hypothesized that increased levels of INS-17 could be sufficient to promote ITAM behavior in the absence of the FD step prior to 1 CS-US pairing. To test this, we examined if wild-type animals fed INS-17 peptide required FD for enhanced ITAM. We compared the learning and memory performance of SCR or INS-17 fed animals either non-FD or FD prior to training, and found that INS-17 fed animals exhibited significantly more robust ITAM than SCR fed animals in the non-FD condition (Figs 6D and S6D-S6E). Furthermore, peptide feeding had no detectable effect on ITAM performance of FD animals (Fig S6F). These results suggest that INS-17 acts as a FD signal to promote a stronger association between a food and an odorant.

We next asked if INS-17 functions generally as a FD signal to promote associative behaviors using an aversive olfactory training paradigm, where an attractive odorant diacetyl is paired with the absence of food to form a negative association [74]. If INS-17 functions as a signal of FD internal state required for associative behaviors, we hypothesized that *ins-17* mutants would have impaired aversive learning and memory compared to wild-type animals, because sensing starvation is required for a negative diacetyl association to be formed. Wild-type and *ins-17* loss-of-function mutants underwent well-established aversive memory assays using a diacetyl-starvation pairing [74]. Strikingly, we found that *ins-17* is required for normal aversive associative behaviors, as *ins-17* animals had severe deficits in learning, STAM, and ITAM behavior compared to wild-type animals (Figs 6E and S6G). The aversive and appetitive olfactory conditioning paradigms we tested here recruit distinct neuronal circuits [63,75–81]. Therefore, our results suggest that *ins-17* may be broadly required in the nervous system, responding rapidly to low nutrient availability to signal a FD state so that the valence of olfactory stimuli (positive when paired with food, negative when paired with starvation) can be processed appropriately to promote advantageous behaviors (Fig 6F). This could explain how IIS acts in the nervous system to integrate metabolic status and regulate specific behavioral outputs, such as learning and memory.

## Discussion

Across species, insulin signaling is pleiotropic. In the worm, the DAF-2 receptor regulates a wide array of phenotypes, including those related to development, lifespan, metabolism, fecundity, cognitive behaviors, and motility, among others. How does a single receptor mediate such physiologically distinct phenotypes, and why is communicating metabolic status important to these different phenotypes? Here we report that some of these pleiotropic actions result from specific ligand-receptor interactions. In this instance, we show that an olfactory memory phenotype is likely mediated by communication of metabolic status via INS-17, an antagonistic ILP with no previously reported role in behavior (to our knowledge). DAF-2 receptor partial loss-of-function mutants have a well-established extended memory phenotype [31], so it is not surprising that a predicted antagonistic ILP regulated these behaviors. However, we were surprised that only a single strong antagonist appeared to be required in our paradigms.

The importance of INS-17 to the extended memory phenotype is supported by our finding that overexpression through feeding is sufficient to recapitulate the memory phenotype of *daf-2* mutants, though only through the 3-hour extension window that is regulated by neuronal *daf-2* [36]. While our results suggest that INS-17 may specifically interact with neuronal DAF-2/IR, possibly with the neuron specific isoform DAF-2c [56,82,83], it is also possible that peptide supplementation more effectively targets the nervous system than other tissues. Either of these results might explain why we do not observe as beneficial of an effect of INS-17 supplementation in aged animals relative to young adults, since *daf-2* cognitive aging phenotypes seem to be primarily mediated by the hypodermis [36]. A better understanding of the fascinating biology of this valuable new tool in the future will provide key insights into these findings. While we established that other antagonists are not required for the early phases of this *daf-2*-specific behavioral phenotype, it is possible that others might act as signals to the hypodermis. Moreover, many ILPs are diverse and act as agonists or antagonists in a context-dependent fashion, and most do not have a described role in associative behaviors. Future studies systemically characterizing these diverse ligands will be informative for defining DAF-2 receptor pleiotropy across tissues.

Interestingly, several transcriptomic datasets show that *ins-17* transcripts are increased roughly 2-fold in *daf-2* mutants and decreased in *daf-16* mutants compared to wild-type animals (S1 Table, [39,49,51,60–62]). This indicates that INS-17 is highly regulated by IIS signaling and is potentially acting both upstream and down-stream of DAF-2/IR. “ILP to ILP” signaling, where the action of one ILP affects the expression of another ILP, could be a component of *ins-17*’s role in integrating sensory cues and nutrient availability to elicit behavioral outputs. One example of “ILP to ILP” signaling in behavior is between INS-6 and INS-7 in aversive pathogen learning, where INS-6 released by the ASI neuron regulates *ins-7* transcription in the URX neurons to regulate this behavior [84]. Further transcriptional analysis of *ins-17* mutants and INS-17 overexpression models could provide insight into which ILPs may be regulating or be regulated by INS-17.

We also report that INS-17’s function in adulthood is potentially specific to behaviors, including roaming, learning, and memory, as manipulating INS-17 does not affect lifespan [49]. While *daf-2* animals’ extended lifespan is a canonical phenotype, there may exist alternate downstream IIS pathways that are dispensable for lifespan regulation. There are reports that the sole insulin receptor substrate homolog IST-1 may differentially activate distinct arms of the IIS signaling pathway, as it can function upstream of—or partially in parallel to—DAF-16 and is not required for normal growth and development [56,85,86]. Thus, it is possible the 40 known ILPs may similarly engage the IIS pathway. In the present study, we investigated IST-1, which engages atypical IIS signaling to regulate aversive olfactory learning [56]. We report that *ist-1* mutants display positive olfactory memory deficits that were rescued by INS-17 peptide feeding; taken with our similar results in *daf-16* mutants, these findings suggest INS-17 may activate multiple arms of the IIS pathway to regulate adult cognitive behaviors. We also find that *daf-16;ist-1* double mutants do not respond to INS-17 peptide supplementation, suggesting that, similar to other olfactory behavioral paradigms [56], INS-17 engages multiple pathways that can act partially in parallel to one another downstream of DAF-2 to regulate behavior. It will be intriguing in the future to examine if other DAF-2-mediated associative behaviors similarly engage both downstream pathways.

We also find that the genomic context in which an ILP is regulated provides specificity, as “swapping” a structurally similar insulin, *ins-37* [47], into *the ins-17* genetic locus is sufficient to functionally replace *ins-17* in behavior, though endogenous *ins-37* is not required for learning phenotypes in our paradigm. One potential caveat to our approach is that we only manipulated the endogenous *ins-17* locus in these animals, and they still expressed an additional endogenous copy of *ins-37*, which could have off target-effects*.* However, behavioral rescue after this CRISPR replacement was comparable to wild-type animals, and sensory function and development were not grossly affected. It is possible that other phenotypes not measured in this study could be altered in this strain. Overall, our CRISPR “swap” result supports that tissue-specific gene regulation may inform DAF-2/IR pleiotropy, at least with regards to behavior, warranting future studies exploring tissue-specific requirements for DAF-2/IR function.

Furthermore, we demonstrate that INS-17 acts to communicate internal metabolic status, and that proper communication of internal metabolic state is required for both positive and aversive associative behaviors. Both associative behavioral paradigms include a relatively short, 1-hour FD step. For appetitive conditioning, this is prior to training to prime animals to form a stronger association between food and an odorant, thus promoting a more robust behavioral response, while for aversive conditioning this FD step is paired with a normally preferred odor to induce aversion. These FD steps occur within the timeframe before animals display significant changes in sensory integration behavior due to a “lack of food sensation”, which begins after 3 hours of food-deprivation [87]; however, food is cleared from the alimentary system within 2 minutes of ingestion [88,89], suggesting the need for a FD signal to inform robust behavioral responses after only 1 hour of FD.

Our gene expression data, similar to previous studies [51,60,61], shows that the *ins-17* gene is activated in this timeframe, suggesting that could it be involved in responding to these brief FD to act as a “signal”. This notion is supported by our findings that feeding INS-17 alone is sufficient to alter roaming behavior, leading animals to dwell more and roam less, consistent with internally sensing a nutrient deprived state [65,66,70] Finally we demonstrated that feeding INS-17, and theoretically mimicking nutrient deprivation internally, to animals that do not undergo FD results in normal learning and memory. This suggests that INS-17 is the signal responsible for the memory-promoting effects of FD. Together, these results suggest that INS-17 could be released in response to acute and prolonged FD to inform sensory integration behavior.

Interestingly, the only phenotype previously associated with INS-17 is dauer formation to protect progeny from unfavorable conditions [49], namely starvation [90]. We speculate that INS-17 may respond to FD to coordinate phenotypic responses in different life stages that are most advantageous; for example, while *ins-17* regulates dauer formation, which is a response to lack of food availability and other stresses during development, it may also regulate the ability to form associations in the context of food availability in adulthood, which is beneficial for egg-laying and progeny survival [71,91]. This dual-role for a metabolic signal in developmental stress responses and behavioral plasticity in the adult worm is not exclusive to *ins-17.* DAF-7, a TGF-beta ligand, regulates dauer formation [92–94], behavioral plasticity in response to stress such as pathogen learning and transgenerational pathogen avoidance [95–97], and is necessary for proper isothermal tracking behavior, another associative behavior [98]. Another essential nutrient sensing pathway, TOR signaling, specifically intestinal Rictor/TORC2, regulates both dauer formation and adult exploratory behavior, through communicating to the nervous system [99]. Rictor/TORC2-dependent intestine-to-neuron communication is also important for gustatory associative learning [100]. Our results add to this body of literature that highlights the importance of considering how altering nutrient status, even for a brief time window, can influence a wealth of behavioral phenotypes. In the future, it will be exciting to study the interplay between these various signals, as well as how other ILPs may be regulated in the context of changing nutrient availability to modulate phenotypic plasticity.

Since *ins-17* is required for positive and aversive associative behaviors, and can regulate roaming behavior, our results support that INS-17 may function as a broad signal for nutrient deprivation to the nervous system. *ins-17* is widely expressed across the nervous system [39,58], but is most highly expressed in the AIZ interneuron, which mediates a variety of behavioral outputs in response to multiple sensory neurons and circuits [101–103]. We observed that associative behavior impairments resulting from loss of *ins-17* do not stem from baseline sensory deficits, which strongly indicates that rather sensory information may not be integrated correctly or that sensory neurons cannot alter their responses to an environmental stimulus in the context of altered metabolic status. We have established that INS-17 may be essential for interpreting internal state and proper valence processing. In mammals, neuropeptides also are important for signaling valence in positive and aversive associative paradigms [104,105], and this is likely a theme of peptides modulating circuits more broadly. Future studies examining how INS-17 regulates sensory circuit activity in various contexts will help further define *daf-2* pleiotropic mechanisms, and how metabolic changes are communicated to regulate behavior.

Our results bring an attractive model for the expansion of the ILP family in *C. elegans*. ILPs are encoded across the genome and function under the control of a variety of regulatory elements so the animal can dynamically respond to a combination of nutrients and environmental stimuli via DAF-2/IR to facilitate the most advantageous phenotype. Having a diverse array of peptides that can carry out distinct outputs is beneficial, especially if there is only one receptor to mediate such outputs. In the case of INS-17, being able to properly make associations in the face of changing environments can promote survival, such as remembering availability (or lack) of a food source associated with specific odors. Together, our findings have identified INS-17 as a physiological signal that is required for positive and aversive associative behaviors and may integrate environmental cues and nutrient status to inform valence processing and regulate behavioral outcomes. This research underscores the importance of future work investigating the links between metabolism and phenotypic plasticity.

## Materials and methods

### General *C. elegans* maintenance

Worms were maintained at 20 °C on plates containing either 1) Standard nematode growth medium (NGM): 3 g/L NaCl, 17 g/L agar, 2.5 g/L peptone, 25 mL/L KPO4 (pH 6.0), 1 mL/L MgSO4, 1 mL/L CaCl2, and 1 mL/L cholesterol (5 mg/mL in ethanol) [106]; or 2) High growth medium (HGM): 3 g/L NaCl, 30 g/L agar, 20 g/L peptone, 25 mL/L KPO4 (pH 6.0), 1 mL/L MgSO4, 1 mL/L CaCl2, and 4 mL/L cholesterol (5 mg/mL in ethanol) [50]. Plates were seeded with an *E. coli* OP50 lawn for ad libitum feeding [106]. Animals were developmentally synchronized using an alkaline-bleach solution (1.5 mL 5 N NaOH, 3.0 mL 5% sodium hypochlorite, 5.5 mL water) to collect eggs from gravid adults; eggs were subsequently washed three times with M9 buffer solution (6 g/L Na2HPO4, 3 g/L KH2PO4, 5 g/L NaCl and 1 mL/L 1M MgSO4 in ultrapure water) before plating [106].

#### Strains.

Wild-type: N2 Bristol. Mutants: *ins-17(tm790), ins-21(tm5180)*, *ins-37(tm6892)*, and *ins-39(tm6520)* were obtained from the National BioResource Project, Japan. RB2489 (*ins-15(ok3444)*), RB2594 (*ins-22(ok3616)*), CB1370 (*daf-2(e1370)*), CF1038 (*daf-16(mu86)*), MAH677 (*sid-1(qt9) V; sqIs71[rgef-2p::GFP + rgef-1p::sid-1]*) were obtained from the Caenorhabditis Genetics Center. CX17790 (*ist-1(ok2706))* was described previously [56] and generously gifted to us by Dr. Cornelia Bargmann. Strains made in collaboration with InVivo Biosystems include *ins-17* CRISPR KO animals with *ins-37* replacement COP2848 (*ins-17(knu1330[ins-37]) III*) and *ins-17* CRISPR KO animals COP2835 (*ins-17(knu1320) III*). The following strains were generated by crosses: RNA34 (*daf-2(e1370)*; *ins-17(tm790))* and RNA59 (*daf-16(mu86); ist-1(ok2706)*).

### Peptide plasmid generation and transformation

The amino acid coding sequence for the INS-17 peptide was identified using www.wormbase.org [58], while the previously published scramble control sequence, which has no homology to any known peptide (C-x-N) is as follows: NSKLHRGGGRSRTSGSTGSMASHARGSPGLQ [53]. These sequences were codon-optimized for bacteria and cloned in pET-21a(+) cloning vector via XhoI/XhoI strategy by GenScript. The scramble control sequence and plasmid were generated using the same cloning strategy by GenScript. Plasmids arrived lyophilized and were re-suspended in nuclease-free H_2_O to a concentration of 100ng/μl and stored at -20 °C. Each expression clone was transformed into competent iOP50 cells using methods previously described [107]. Briefly, iOP50 stocks were streaked out onto LB plates containing 50 μg/mL tetracycline and grown at 37 °C overnight. Then, single colonies of iOP50 were isolated and used to inoculate 2.0 mL LB containing 50 μg/ml tetracycline and placed in a 37 °C shaker overnight. 0.5 mL of each culture was used to inoculate 3.0 mL of LB without antibiotics and grown for 2 hours in a 37 °C shaker. Each culture was then aliquoted in 0.75 mL increments into four 1.5 mL tubes and cooled for 10 minutes on ice. Next, the cells were harvested by centrifugation at 6,000 rpm for 5 minutes at 4 °C. The resulting supernatant was discarded, and cells were placed back on ice and re-suspended in 1.0 mL 100 mM ice-cold CaCl_2_ solution. Cells were kept on ice for 20 minutes and then harvested by centrifugation as in the previous step. After discarding the supernatant, cells were re-suspended in 150μl of ice-cold CaCl_2_ solution and placed on ice to be immediately used for transformation.

Competent cells were transformed using the New England Biolabs protocol (C2987H/C2987I). Single colonies of transformed cells were isolated and cultured in 3.0 mL of LB containing 50 μg/mL tetracycline and 50 μg/mL carbenicillin overnight in a 37 °C shaker. Cultures were frozen down as glycerol stocks and stored at -80 °C. Successful transformation was verified by isolating plasmids from cultures using a Zyppy Plasmid Miniprep Kit and sending samples for whole-plasmid sequencing (Plasmidsaurus).

### Peptide plate preparation

LB containing 50 μg/mL tetracycline and 50 μg/mL carbenicillin was inoculated with iOP50 encoding either the peptide sequence of interest or scramble peptide sequence and cultured for 18–20 hours in a 37 °C shaker. 100 mm NGM plates containing 50 μg/mL tetracycline, 50 μg/mL carbenicillin, and 1.0 mL/L isopropyl-β-D-thiogalactoside (IPTG) were plated with 1.0 mL of corresponding culture. Seeded plates were left to dry at least 24 hours at room temperature and never kept longer than 72 hours before use. Approximately one hour before transferring worms to peptide or scramble control plates, 200 μL of 0.1 M IPTG was added to each plate and left to dry.

### Peptide feeding

We adapted a previously published peptide feeding-based approach [53]. Worms were maintained on NGM agar plates seeded with OP50 *E. coli* and maintained at 20 °C until the start of experiments. All animals were transferred to a conical tube and washed three times with M9 buffer solution before being transferred to NGM plates containing 50 μg/mL Tetracycline, 50 μg/mL Carbenicillin, and 1.0 mM IPTG that were seeded with 1.0 mL of iOP50 expressing either the scramble peptide or the peptide of interest. Animals were kept on the scramble or peptide-expressing lawns for at least 48 hours of exposure time at 20 °C before performing assays. Exact peptide feeding timeline parameters are expounded in the results section.

### RNAi treatment

Worms were maintained on NGM agar plates seeded with OP50 *E. coli* at 20 °C until the start of experiments. At the L4 stage, animals were transferred to a conical tube and washed three times with M9 buffer solution before being transferred to NGM plates containing 50 μg/mL Carbenicillin and 1.0 mM IPTG that were seeded with 1.0 mL of HT115 bacteria expressing either RNAi against the gene of interest or the vector control RNAi. Animals were kept on the RNAi lawns for 48 hours of exposure time at 20 °C before performing assays at Day 2 of adulthood.

### Naïve chemotaxis assay

Chemotaxis assays were performed using a protocol based on previously published assays [108]. Briefly, assays were performed on unseeded 100mm NGM plates. On the back of each plate, two marks were made on opposite sides of the plate, approximately 5mm from the edge. 1μL of sodium azide (Thermo Fisher) was placed on both spots and allowed to dry before adding 1μL of odorant (Sigma Aldrich) diluted in ethanol on one mark and 1μL ethanol on the other. Using M9 buffer, worms were washed off their plates and subsequently washed three times to eliminate any leftover bacteria that could impact baseline behavior. Then, worms were placed near the bottom center of the plate, equidistant between the two marks, and allowed to chemotax for an hour. Chemotaxis indices were calculated per plate as follows:


Chemotaxis Index=(# of worms butanone−# of worms ethanol)/(total # of worms)

For naïve chemotaxis assays performed to assess baseline chemosensory function, we tested the naïve preference to a highly appetitive concentration of butanone (0.1%) as previously reported [108].

### Positive associative butanone learning and memory assay

Worms were trained and tested for learning, short- and intermediate-term memory changes as previously described [50]. Briefly, synchronized adult worms were washed off plates with M9 buffer and allowed to settle by gravity, followed by two subsequent washes with M9 buffer to remove leftover bacteria that could alter baseline behavior. Next, the worms were food-deprived for one hour in M9 buffer before exposure to 1 CS-US pairing, where food-deprived worms were transferred to 100mm NGM conditioning plates seeded with OP50 *E*. *coli* bacteria (or iOP50 bacteria containing a peptide or scramble control plasmid for peptide feeding experiments) and had 16μL of 10% butanone (Sigma Aldrich) diluted in ethanol streaked on the lid in a ‘#’ shape for one hour or for only 15 minutes for sub-threshold conditioning. After conditioning, the trained population of worms were tested for chemotaxis to 10% butanone and to an ethanol control using standard, previously described chemotaxis conditions [108]. Different stages of memory were tested by measuring chemotaxis indices of separate populations of worms at timepoints including 0 min. (learning), 0.5 hour (STAM), 1 hour (ITAM) and 2 hours (forgetting) after training. For timepoints after learning behavior, worms were transferred to hold plates seeded with OP50 *E. coli* or iOP50 bacteria for peptide feeding experiments as stated above. Chemotaxis indices for each timepoint were calculated as detailed in “Naïve chemotaxis assays” section.

Performance indices for each biological replicate (total of 3 replicates, 5 plates/replicate, at least 50–100 worms/plate) is calculated relative to the average naïve chemotaxis index for behavior on that day; it is measured as the change in the chemotaxis index after training relative to the average naïve (untrained) chemotaxis index:


$$𝐏𝐞𝐫𝐟𝐨𝐫𝐦𝐚𝐧𝐜𝐞\;𝐢𝐧𝐝𝐞𝐱={\text{Chemotaxis index}}_{\text{trained}}-\mu [{\text{Chemotaxis index}}_{\text{untrained}}]$$

% Maximum Performance Indices are performance indices represented as a percentage of the maximum value that can be performed relative to the naïve chemotaxis index [31,35]. This % can be more informative for visualizing performance indices when naïve chemotaxis behavior is high, as in *daf-2(e1370)* animals.

This is calculated for each timepoint by dividing the performance index of a timepoint by the average of the untrained chemotaxis indices subtracted from 1 and multiplying by 100:


$$\%\;{𝐌𝐚𝐱𝐢𝐦𝐮𝐦\;𝐏𝐞𝐫𝐟𝐨𝐫𝐦𝐚𝐧𝐜𝐞\;𝐈𝐧𝐝𝐞𝐱}_{𝐭𝐢𝐦𝐞𝐩𝐨𝐢𝐧𝐭}=({\text{Performance index}}_{\text{timepoint}}/(\text{1}-\mu [{\text{Chemotaxis index}}_{\text{untrained}}]))*\text{100}.$$

Finally, rate of memory decay was calculated for wild-type animals and *ins-17(tm790)* animals to separate potential perceived memory impairments from learning impairments. The equation calculates the change in % maximum performance for a given timepoint relative to learning behavior (0hr or T0) performance. The equation is depicted below:


$$𝐌𝐞𝐦𝐨𝐫𝐲\;𝐃𝐞𝐜𝐚𝐲=\;\Delta \text{T}\text{0}\;\%\;{\text{Maximum Performance Index}}_{\text{timepoint }}=(({\text{PI}}_{\text{timepoint}}\;-{\text{T0}}_{\text{avg}})/\;{\text{T0}}_{\text{avg}})*\text{100}$$

### Butanone enhancement assay

This assay was adapted from previously described methods [63]. Unlike the positive butanone associative memory assays, butanone enhancement assays skip the 1-hour food-deprivation step prior to the conditioning step. All other methods are identical to those listed in the positive butanone associative memory assay section.

### Negative associative learning and memory assay

Worms were tested for learning and memory changes in response to aversive diacetyl training, adapted from previously published methods [74]. Similar to positive associative assays, synchronized Day 1 adult worms were washed off plates with M9 buffer and then allowed to settle by gravity and washed twice more with M9 buffer. After washing, the worms were conditioned for one hour by food-deprivation on 100mm NGM plates with no bacteria and 16uL of 100% diacetyl (Sigma Aldrich) streaked on the lid in a ‘#’ shape. The trained population of worms were then tested for chemotaxis to 1% diacetyl and to an ethanol control using standard, previously described chemotaxis conditions [108]. Learning and memory performance indices were calculated for each timepoint as previously stated in the positive butanone associative assay section.

### Roaming assay

Roaming assays were performed by transferring a single L4 animal to a 35mm petri dish with NGM solid media seeded with an OP50 bacterial lawn that was allowed to dry for 24 hours prior to transfer, similar to methods previously described [67]. Animals were left to roam for 16–20 hours and then worm tracks were counted by superimposing a 25mm x 25mm grid underneath the plate and recording the number of squares that overlapped with the tracks.

### Lifespan assay

Lifespan assays were performed on *ins-17(tm790)* animals and wild-type animals that were bleached onto NGM plates seeded with OP50 *E. coli* and maintained at 20 °C; at the L4 larval stage, worms were picked for survival analysis and transferred every other day to freshly seeded plates. Similarly, lifespan assays with peptide feeding administration were performed on wild-type animals that were bleached onto NGM plates seeded with OP50 *E. coli* and maintained at 20 °C. Then, at the L4 larval stage, worms were picked for survival analysis and transferred every other day to NGM plates containing 50 μg/mL tetracycline, 50 μg/mL carbenicillin, and 1.0 mL/L IPTG that were seeded with either INS-17 iOP50 or scramble iOP50 *E. coli*. Lifespans began (t = 0) at Day 1 of adulthood and had an n = ~120 worms per strain/trial for a total of three biological replicates per assay.

### Food-deprivation behavioral experiments

For food-deprivation experiments, well-fed, synchronized adult worms were washed off plates using M9 buffer into a 15mL conical and washed two more times to remove bacteria that could alter baseline behavior. Next, worms were either 1) food-deprived for one hour on a 100mm NGM plate without food or 2) non-food deprived for one hour by being transferred to a 100mm plate seeded with a bacterial lawn (iOP50 bacteria for peptide feeding experiments). We transferred worms to solid media plates without food instead of food-depriving them in M9 buffer solution (as in our typical behavioral paradigm) to control for any potential changes that could occur as a result of moving between solid and liquid media for only one condition (food-deprived). Worms were then immediately exposed to the conditioning step (1 CS-US pairing) and stages of learning and memory were tested as stated in the positive associative butanone learning and memory assays section.

### RNA isolation, cDNA synthesis and qRT-PCR

For qRT-PCR food-deprivation experiments, well-fed, synchronized adult worms were washed off plates using M9 buffer into a 15mL conical and washed two more times to remove bacteria that could alter baseline behavior. Next, worms were either 1) food-deprived for one hour or two hours after being transferred to a 100mm plate with no bacterial lawn or 2) non-food deprived for one hour or two hours after being transferred to a 100mm plate seeded with a bacterial lawn.

After feeding or food deprivation, worms were crushed in liquid nitrogen using a mortar and pestle and added to Trizol (Thermo Fisher Scientific). RNA was isolated per manufacturer’s instructions, followed by DNase treatment (Qiagen). cDNA was synthesized with an oligo dT primer and Superscript III reverse transcriptase enzyme (Thermo Fisher Scientific). cDNA was mixed with buffers, primers, SYBR green, and hot start Taq polymerase in a master mix prepared by a manufacturer (Thermo Fisher Scientific). PCR reactions were run using a Quant Studio 7 Pro Dx Real-Time PCR System (Thermo Fisher Scientific), followed by a dissociation reaction to determine specificity of the amplified product. Gene expression was quantified using the ΔΔCt method using *pmp-3* as a reference gene which is not responsive to starvation nor multiple other stresses and it maintains constant expression levels in the context of manipulating insulin signaling [109,110]. Primer sets were as follows:

*ins-17* For: 5’- GGACACTATCCGACCACCAC -3’

*ins-17* Rev: 5’- TTCACATCCCGTCTCACAGC -3’

*pmp-3* For: 5’- AGTTCCGGTTGGATTGGTCC -3’

*pmp-3* Rev: 5’- CCAGCACGATAGAAGGCGAT-3’

### Statistical analysis and software

All statistics (excluding survival analysis) were calculated with GraphPad Prism Software. All statistical data are reported in the main text, figures, and tables. Significance threshold of p < 0.05 was used. The symbols *, **, ***, and **** refer to p < 0.05, 0.01, 0.001, and 0.0001. The symbols #, ##, ###, and #### refer to p < 0.05, 0.01, 0.001, and 0.0001. For comparing performance indices between two behavior conditions (e.g., *vector control* vs *ins-17 RNAi*), we used a Mann-Whitney test comparing ranks, as it does not assume normality. For comparing performance indices between three or more groups (e.g., wild-type vs multiple insulin receptor antagonist mutants), we performed one-way analysis of variances followed by Bonferroni post hoc tests for multiple comparisons. Two-way ANOVAs were used for evaluating effects between genotype and/or treatment and timepoint (T0, T30, T60, T120) on performance indices with a significant interaction between factors (p < 0.0001) that prompted Bonferroni post-hoc analyses to determine differences between individual groups. Sample size n represents the number of chemotaxis assays performed for behavioral experiments, with each assay containing approximately 50–150 worms. All experiments were performed on different days with distinct populations to confirm reproducibility. For qPCR analysis, unpaired t-tests with Welch’s corrections were performed to determine significance between conditions. Survival analysis statistics were generated using the log-rank (Mantel-Cox) method to test the null hypothesis in Kaplan-Meier survival analysis and evaluated using OASIS survival analysis software [111].

## Supporting information

S1 Fig *daf-2(e1370)* animals and *ins-22(ok3616)* animals have higher baseline naïve chemotactic preference for 10% butanone. **(A)** *daf-2(e1370)* animals have a higher baseline naïve chemotaxis to 10% butanone compared to wild-type animals. Mann-Whitney test comparing ranks. Mean ± SEM. n = 10 per genotype. ***p < 0.001**. (B)** Raw performance indices for wild-type animals decline at two hours compared to *daf-2(e1370)* mutants at six hours after 1 CS-US pairing. Mean ± SEM. n = 10 per genotype. **(C)** *ins-17(tm790)* animals have no detectable impairments in baseline naïve chemotaxis to the neutral concentration of butanone (10%) **(D)** nor the appetitive concentration of butanone (0.1%) compared to wild-type. Mann-Whitney test comparing ranks. Mean ± SEM. n = 15 per genotype. ns, not significant (p > 0.05). **(E)** Neuron-specific RNAi treatment has no detectable effect on naïve chemotaxis to 10% butanone nor **(F)** 0.1% butanone. Mann-Whitney test comparing ranks. Mean ± SEM. n = 15 per genotype. ns, not significant (p > 0.05). **(G)** Neuron-specific RNAi treatment for *ins-17* results in significant memory decay at the STAM and ITAM timepoints compared to wild-type decay at these timepoints. Two-way ANOVA with Bonferroni’s multiple comparisons test. Interaction between factors, p = 0.0001; timepoint, p < 0.0001; genotype, p < 0.0001. Mean ± SEM. n = 15 per genotype. ***p < 0.001, ****p < 0.0001. **(H)** Weak (*ins-15, ins-21*) and **(I)** strong (*ins-39*) insulin receptor antagonist mutants have no detectable deficits in baseline naïve chemotaxis to 10% butanone. Strong antagonist *ins-37* has a slight impairment compared to wild-type, but this deficit does not appear to affect learning and memory behaviors. One-way ANOVA with Bonferroni’s multiple comparisons test (**S1H Fig**, ns; **S1I Fig,** p < 0.05). Mean ± SEM. n = 15 per genotype. *p < 0.05; ns, not significant (p > 0.05). **(J)** Weak and **(K)** strong antagonist mutants have normal learning behavior compared to wild-type after 1 CS-US pairing. One-way ANOVA with Bonferroni’s multiple comparisons test (p > 0.05, ns). Mean ± SEM. n = 15 per genotype. ns, not significant (p > 0.05). **(L)** *ins-22(ok3616)* animals have higher naïve attraction to 10% butanone that impair the ability to measure meaningful deficits in learning and memory behaviors. Mann-Whitney test comparing ranks. Mean ± SEM. n = 15 per genotype. ***p < 0.001.
(TIFF)

S2 Fig Peptide administration (SCR and INS-17) does not affect baseline naïve attraction to 0.1% nor 10% butanone. **(A)** *daf-2(e1370)* animals have a higher baseline naïve chemotaxis to 10% butanone compared to *ins-17(tm790)* and *daf-2(e1370)*; *ins-17(tm790)* animals. This higher naïve preference appears to contribute to **(B)** *daf-2(e1370)* learning and **(C)** 3hr-memory deficits compared to *daf-2(e1370)*; *ins-17(tm790)* double mutants, but we find this significance is lost when comparing % Max Performance Indices as in **Fig 2A**. One-way ANOVA with Bonferroni’s multiple comparisons tests (**S2A Fig**, p < 0.0001; **S2B Fig**, p < 0.01; **S2C Fig**, p < 0.0001). Mean ± SEM. n = 15 per genotype. *p < 0.05, **p < 0.01, ****p < 0.0001; ns, not significant (p > 0.05). **(D)** SCR and INS-17 peptide treatments have no detectable effects on naïve chemotaxis behavior of wild-type worms nor *ins-17* mutants to 10% butanone as well as **(E)** 0.1% butanone. One-way ANOVA with Bonferroni’s multiple comparisons tests (p > 0.05, ns). Mean ± SEM. n = 10–15 per genotype. ns, not significant (p > 0.05). **(F)** Naïve chemotaxis and **(G)** learning performance indices corresponding to **Fig 2D.** Mann-Whitney test comparing ranks. Mean ± SEM. n = 15 per genotype. ns, not significant (p > 0.05). **(H)** Wild-type animals fed SCR or INS-17 have no significant differences in learning behavior after a sub-threshold conditioning period of 15 minutes. Mann-Whitney test comparing ranks. Mean ± SEM. n = 15 per genotype. ns, not significant (p > 0.05). **(I)** SCR and INS-17 peptide treatments have no detectable effects on naïve chemotaxis behavior of wild-type worms nor *daf-2(e1370)* animals to 10% butanone. One-way ANOVA with Bonferroni’s multiple comparisons tests (p < 0.01). Mean ± SEM. n = 15 per genotype. ns, not significant (p > 0.05). **(J)** *daf-2(e1370)* extended memory performance is slightly enhanced by INS-17 peptide feeding compared to SCR fed controls at the 6-hour timepoint. Two-way ANOVA with Bonferroni’s multiple comparisons test. Interaction between factors, p = 0.1143; timepoint, p, 0.0001; genotype/treatment, p = 0.0237. Mean ± SEM. n = 15 per genotype. **p < 0.01; ns, not significant (p > 0.05).
(TIFF)

S3 Fig *ins-17(tm790)* animals and manipulation of INS-17 levels via peptide administration have no detectable changes in lifespan compared to wild-type animals and SCR fed worms. **(A)** Wild-type animals and *daf-2(e1370)* mutants have increased naïve chemotaxis to 10% butanone with age. Two-way ANOVA with Bonferroni’s multiple comparisons test. Interaction between factors, p = 0.6087; timepoint, p < 0.0001; genotype, p = 0.0119. Mean ± SEM. n = 10 per genotype. ns, not significant (p > 0.05). **(B)** *daf-2(e1370)* mutants maintain learning and **(C)** ITAM abilities better than wild-type animals with age across Days 1, 3 and 5 of adulthood. Two-way ANOVA with Bonferroni’s multiple comparisons test. For **S3B**, interaction between factors, p = 0.2450; timepoint, p = 0.0032; genotype, p = 0.0016. For **S3C**, interaction between factors, p = 0.0573; timepoint, p = 0.0020; genotype, p < 0.0001. Mean ± SEM. n = 10–15 per genotype. *p < 0.05, ****p < 0.0001; ns, not significant (p > 0.05). **(D)** Wild-type animals treated with SCR or INS-17 at either the L4 stage or at **(E)** Day 3 of adulthood had no detectable phenotypic effects in naïve chemotaxis to 10% butanone at Day 5 of adulthood. Mann-Whitney test comparing ranks. Mean ± SEM. n = 15 per genotype. ns, not significant (p > 0.05). **(F-G)** Replicates 2 and 3 of data represented in **Fig 3G**. Probability of survival analysis demonstrates there is no significant difference in lifespan comparing wild-type animals and *ins-17(tm790)* animals. Mean/median ± SEM. n ≥ 100 per replicate. ns, not significant (p > 0.05). **(H-I)** Replicates 2 and 3 of data represented in **Fig 3H.** Probability of survival analysis for wild-type animals treated with SCR peptide compared to wild-type animals fed INS-17 peptide shows no significant difference in average lifespan. Mean/median ± SEM. n ≥ 100 per replicate. ns, not significant (p > 0.05).
(TIFF)

S4 Fig *daf-16(mu86);ist-1(ok2706)* demonstrate significant memory deficits likely not due to slight sensory deficits in baseline attraction to appetitive butanone. **(A)** Wild-type animals and *daf-16(mu86)* mutants have naïve chemotaxis behavior to 10% butanone that is not significantly different. Mann-Whitney test comparing ranks. Mean ± SEM. n = 10 per genotype. ns, not significant (p > 0.05). **(B)** SCR and INS-17 peptide treatments have no detectable effects on naïve chemotaxis behavior of wild-type worms nor *daf-16* mutants to 10% butanone. One-way ANOVA with Bonferroni’s multiple comparisons tests (p < 0.0001). Mean ± SEM. n = 15 per genotype. ns, not significant (p > 0.05). **(C)** Confirmation for experiments in **Fig 4B** that INS-17 peptide feeding resulted in wild-type animals fed INS-17 to display memory behavior at two hours compared to animals fed SCR, which do not display memory behavior at two hours (forgetting). Two-way ANOVA with Bonferroni’s multiple comparisons test. Interaction between factors, p = 0.0058; timepoint, p < 0.0001; treatment, p < 0.0001. Mean ± SEM. n = 15 per genotype. **p < 0.01, ****p < 0.0001; ns, not significant (p > 0.05). **(D)** Wild-type animals and *ist-1(ok2706)* mutants have naïve chemotaxis behavior to 10% butanone that is not significantly different. Mann-Whitney test comparing ranks. Mean ± SEM. n = 15 per genotype. ns, not significant (p > 0.5). Additionally, **(E)** *ist-1(ok2706)* naïve chemotaxis to attractive 0.1% butanone, while significantly higher than wild-type, is not impaired. Mann-Whitney test comparing ranks. Mean ± SEM. n = 10 per genotype. **p < 0.01. **(F)** SCR and INS-17 peptide treatments have no detectable effects on naïve chemotaxis behavior of wild-type worms nor *ist-1* mutants to 10% butanone. One-way ANOVA with Bonferroni’s multiple comparisons test (p > 0.05, ns). Mean ± SEM. n = 15 per genotype. ns, not significant (p > 0.05). **(G)** Confirmation for experiments in **Fig 4D** that INS-17 peptide feeding resulted in wild-type animals fed INS-17 to display memory behavior at two hours compared to animals fed SCR, which do not display memory behavior at two hours (forgetting). Two-way ANOVA with Bonferroni’s multiple comparisons test. Interaction between factors, p < 0.0001; timepoint, p < 0.0001; treatment, p < 0.0001. Mean ± SEM. n = 15 per genotype. *p < 0.05, ****p < 0.0001; ns, not significant (p > 0.05). **(H)** While *daf-16(mu86); ist-1(ok2706)* double mutants have lowered baseline naïve chemotaxis to the appetitive concentration of butanone (0.1%), these animals do demonstrate attraction to appetitive butanone compared to the neutral concentration as shown in **(I).** This suggests that double mutants do not have sensory deficits in attraction to butanone that would prevent them from forming a positive association with the odorant. Mann-Whitney test comparing ranks. Mean ± SEM. n = 15 per genotype. ***p < 0.001. **(I)** SCR and INS-17 peptide treatments have no detectable effects on naïve chemotaxis behavior of wild-type worms, *daf-16(mu86)*, *ist-1(ok2706)*, and *daf-16(mu86); ist-1(ok2706)* mutants to 10% butanone. Two-way ANOVA with Bonferroni’s multiple comparisons test. Interaction between factors, p = 0.4675; treatment, p = 0.2638; genotype, p < 0.0001. Mean ± SEM. n = 15 per genotype. ns, not significant (p > 0.05). **(J)** Performance indices for STAM and **(K)** forgetting timepoints corresponding to experiments shown in **Fig 4E-4F**. At the T30 timepoint, *ist-1(ok2706)* animals and *daf-16(mu86)* animals fed INS-17 had better memory performance compared to SCR-fed controls, while *daf-16(mu86);ist-1(ok2706)* double mutants displayed no memory behavior. Additionally, wild-type animals and *ist-1(ok2706)* animals fed INS-17 display memory behavior at 2hrs, confirming that peptide feeding worked effectively for these experiments. Two-way ANOVA with Bonferroni’s multiple comparisons test. For **S4J**, interaction between factors, p < 0.0001; treatment, p = 0.0015; genotype, p < 0.0001. For **S4K,** For **S4J**, interaction between factors, p = 0.0411; treatment, p < 0.0001; genotype, p < 0.0001. Mean ± SEM. n = 15 per genotype. ***p < 0.001, ****p < 0.0001; ns, not significant (p > 0.05).
(TIFF)

S5 Fig CRISPR replacement of *ins-37* at *ins-17* genetic locus does not affect chemosensation and *ins-17* expression is upregulated in response to food deprivation but not conditioning. **(A)** There are no detectable differences in naïve chemotaxis to 10% butanone in wild-type animals, *ins-17* CRISPR KO animals (*ins-17(knu1320)*), nor *ins-17p::ins-37* (*ins-17(knu1330[ins-37])*) animals. One-way ANOVA with Bonferroni’s multiple comparisons test (p > 0.05, ns). Mean ± SEM. n = 15 per genotype. ns, not significant (p > 0.05). **(B)** CRISPR KO of *ins-17* results in significant memory decay at the ITAM timepoint compared to wild-type and *ins-17p::ins-37* behavioral decay at this timepoint. Two-way ANOVA with Bonferroni’s multiple comparisons test. Interaction between factors, p = 0.0002; timepoint, p < 0.0001; genotype, p = 0.0005. Mean ± SEM. n = 15 per genotype. * is performance compared to wild-type and # is performance relative to *ins-17p::ins-37* performance. ****/####p < 0.0001. **(C)** qRT-PCR of *ins-17* mRNA levels in Day 1 adult wild-type animals show that two hours of food-deprivation results in increased *ins-17* transcript levels. The dotted line represents a fold-change of 1 relative to the average of non-FD control values, indicating no change between conditions at that level. Mann-Whitney test comparing ranks. Mean ± SEM. n = 5 per genotype. *p < 0.05. **(D)** Untrained wild-type animals have *ins-17* transcript levels not significantly different from animals that underwent the FD step prior to training. The dotted line represents a fold-change of 1 relative to the average of untrained, non-FD control values, indicating no change between conditions at that level. Mann-Whitney test comparing ranks. Mean ± SEM. n = 5 per genotype. ns, not significant (p > 0.5).
(TIFF)

S6 Fig Food-deprivation prior to 1 CS-US pairing does not affect learning nor STAM behavioral performance. **(A)** Wild-type animals FD or non-FD prior to training showed no significant differences between learning and **(B)** STAM behaviors. Mann-Whitney test comparing ranks. Mean ± SEM. n = 15 per genotype. ns, not significant (p > 0.05). **(C)** Food-deprivation prior to training (1 CS-US pairing) does not enhance *daf-2(e1370)* learning performance. Mann-Whitney test comparing ranks. Mean ± SEM. n = 15 per genotype. ns, not significant (p > 0.05). **(D)** Wild-type animals treated with either SCR or INS-17 peptide had learning and **(E)** STAM behaviors not significantly different between the FD SCR or INS-17 fed groups and the non-FD SCR or INS-17 fed groups. One-way ANOVA with Bonferroni’s multiple comparisons test (p > 0.05, ns). Mean ± SEM. n = 10–15 per genotype. ns, not significant (p > 0.05). Meanwhile, **(F)** FD wild-type animals fed SCR had ITAM performance similar to INS-17 fed counterparts. Mann-Whitney test comparing ranks. Mean ± SEM. n = 10–15 per genotype. ns, not significant (p > 0.05). **(G)** *ins-17(tm790)* animals have no detectable deficits in chemotaxis to the highly attractive 1% diacetyl compared to wild-type animals. Mann-Whitney test comparing ranks. Mean ± SEM. n = 10 per genotype. ns, not significant (p > 0.05).
(TIFF)

S1 Table *ins-17* transcripts increase in response to multiple starvation paradigms and genetic models of nutrient deprivation. (TIFF)

S1 Data Source data for all of the main figures. (XLSX)

S2 Data Source data for all of the supplemental figures. (XLSX)
