# Supplementary figures and images for: INS-17 acts as a nutrient deprivation signal to mediate adult IIS-regulated associative behaviors in C. elegans
Source: PLoS Genet. 2026 Apr 28;22(4):e1012130. doi: 10.1371/journal.pgen.1012130 (PMC13143180; doi:10.1371/journal.pgen.1012130)

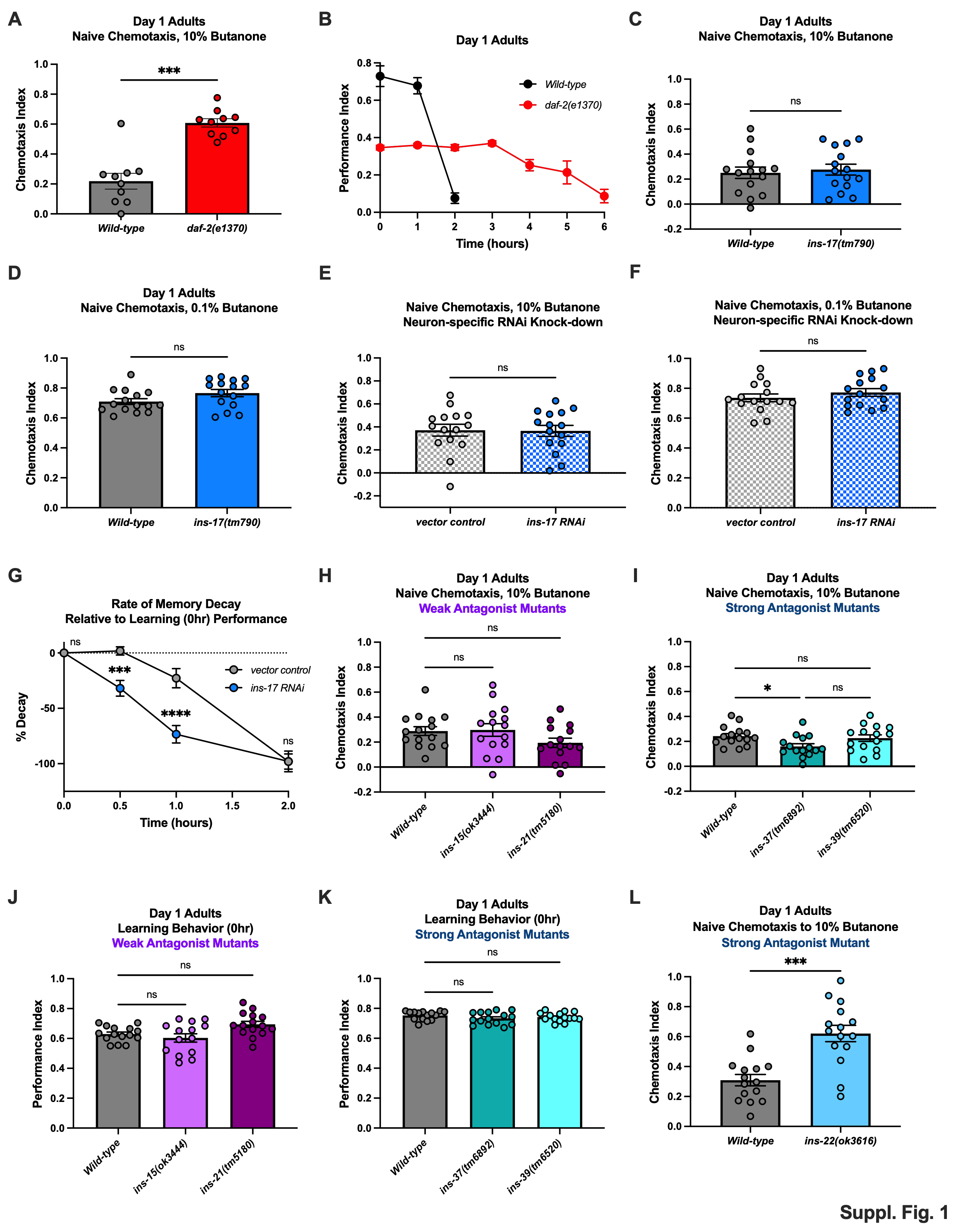

Supplement: S1 Fig — (A) daf-2(e1370) animals have a higher baseline naïve chemotaxis to 10% butanone compared to wild-type animals. Mann-Whitney test comparing ranks. Mean ± SEM. n = 10 per genotype. ***p < 0.001. (B) Raw performance indices for wild-type animals decline at two hours compared to daf-2(e1370) mutants at six hours after 1 CS-US pairing. Mean ± SEM. n = 10 per genotype. (C) ins-17(tm790) animals have no detectable impairments in baseline naïve chemotaxis to the neutral concentration of butanone (10%) (D) nor the appetitive concentration of butanone (0.1%) compared to wild-type. Mann-Whitney test comparing ranks. Mean ± SEM. n = 15 per genotype. ns, not significant (p > 0.05). (E) Neuron-specific RNAi treatment has no detectable effect on naïve chemotaxis to 10% butanone nor (F) 0.1% butanone. Mann-Whitney test comparing ranks. Mean ± SEM. n = 15 per genotype. ns, not significant (p > 0.05). (G) Neuron-specific RNAi treatment for ins-17 results in significant memory decay at the STAM and ITAM timepoints compared to wild-type decay at these timepoints. Two-way ANOVA with Bonferroni’s multiple comparisons test. Interaction between factors, p = 0.0001; timepoint, p < 0.0001; genotype, p < 0.0001. Mean ± SEM. n = 15 per genotype. ***p < 0.001, ****p < 0.0001. (H) Weak (ins-15, ins-21) and (I) strong (ins-39) insulin receptor antagonist mutants have no detectable deficits in baseline naïve chemotaxis to 10% butanone. Strong antagonist ins-37 has a slight impairment compared to wild-type, but this deficit does not appear to affect learning and memory behaviors. One-way ANOVA with Bonferroni’s multiple comparisons test (S1H Fig, ns; S1I Fig, p < 0.05). Mean ± SEM. n = 15 per genotype. *p < 0.05; ns, not significant (p > 0.05). (J) Weak and (K) strong antagonist mutants have normal learning behavior compared to wild-type after 1 CS-US pairing. One-way ANOVA with Bonferroni’s multiple comparisons test (p > 0.05, ns). Mean ± SEM. n = 15 per genotype. ns, not significant (p > 0.05). [file pgen.1012130.s001.tiff]

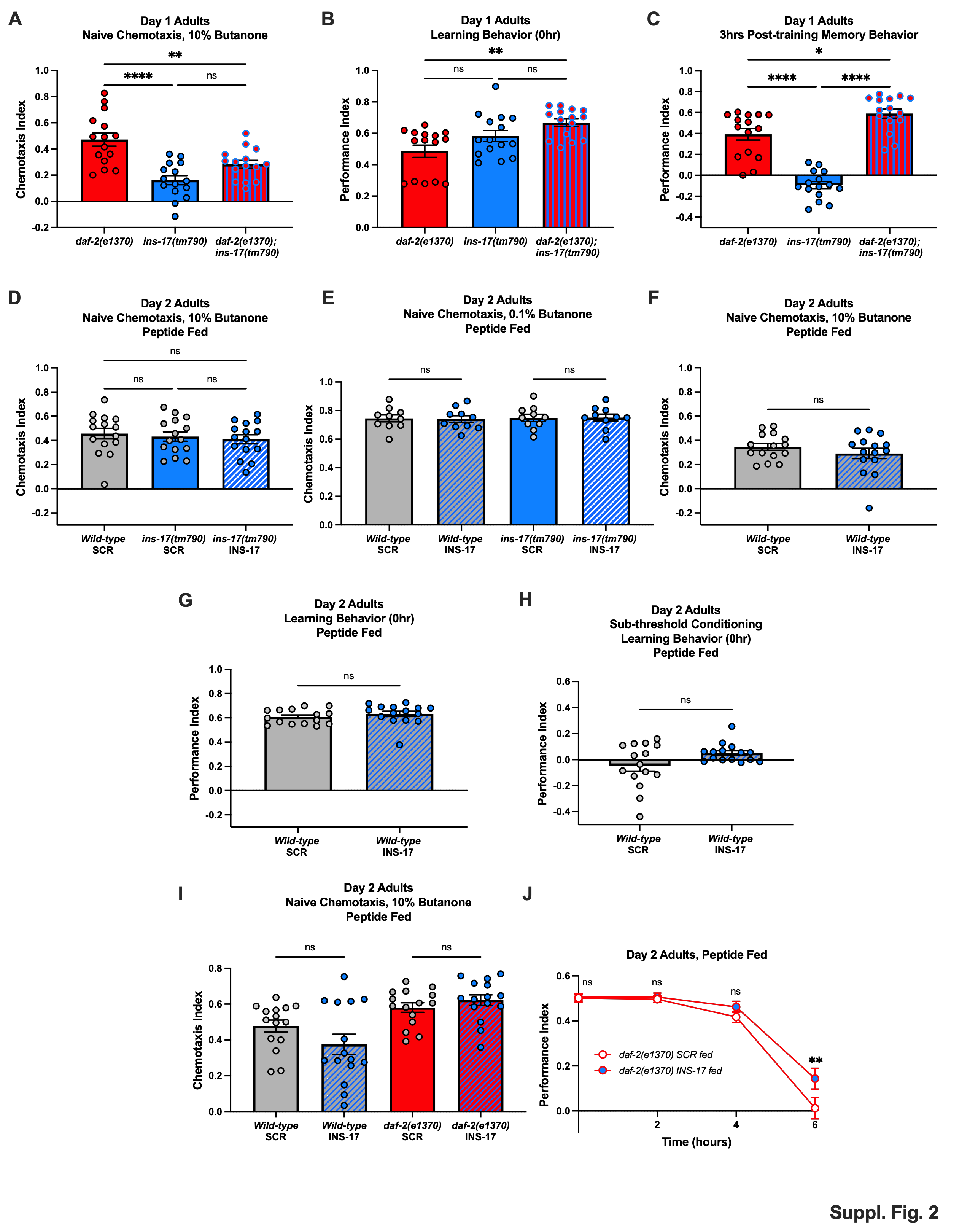

Supplement: S2 Fig — (A) daf-2(e1370) animals have a higher baseline naïve chemotaxis to 10% butanone compared to ins-17(tm790) and daf-2(e1370); ins-17(tm790) animals. This higher naïve preference appears to contribute to (B) daf-2(e1370) learning and (C) 3hr-memory deficits compared to daf-2(e1370); ins-17(tm790) double mutants, but we find this significance is lost when comparing % Max Performance Indices as in Fig 2A. One-way ANOVA with Bonferroni’s multiple comparisons tests (S2A Fig, p < 0.0001; S2B Fig, p < 0.01; S2C Fig, p < 0.0001). Mean ± SEM. n = 15 per genotype. *p < 0.05, **p < 0.01, ****p < 0.0001; ns, not significant (p > 0.05). (D) SCR and INS-17 peptide treatments have no detectable effects on naïve chemotaxis behavior of wild-type worms nor ins-17 mutants to 10% butanone as well as (E) 0.1% butanone. One-way ANOVA with Bonferroni’s multiple comparisons tests (p > 0.05, ns). Mean ± SEM. n = 10–15 per genotype. ns, not significant (p > 0.05). (F) Naïve chemotaxis and (G) learning performance indices corresponding to Fig 2D. Mann-Whitney test comparing ranks. Mean ± SEM. n = 15 per genotype. ns, not significant (p > 0.05). (H) Wild-type animals fed SCR or INS-17 have no significant differences in learning behavior after a sub-threshold conditioning period of 15 minutes. Mann-Whitney test comparing ranks. Mean ± SEM. n = 15 per genotype. ns, not significant (p > 0.05). (I) SCR and INS-17 peptide treatments have no detectable effects on naïve chemotaxis behavior of wild-type worms nor daf-2(e1370) animals to 10% butanone. One-way ANOVA with Bonferroni’s multiple comparisons tests (p < 0.01). Mean ± SEM. n = 15 per genotype. ns, not significant (p > 0.05). (J) daf-2(e1370) extended memory performance is slightly enhanced by INS-17 peptide feeding compared to SCR fed controls at the 6-hour timepoint. Two-way ANOVA with Bonferroni’s multiple comparisons test. Interaction between factors, p = 0.1143; timepoint, p, 0.0001; genotype/treatment, p = 0.0237. Mean ± SEM. n = 15 per g [file pgen.1012130.s002.tiff]

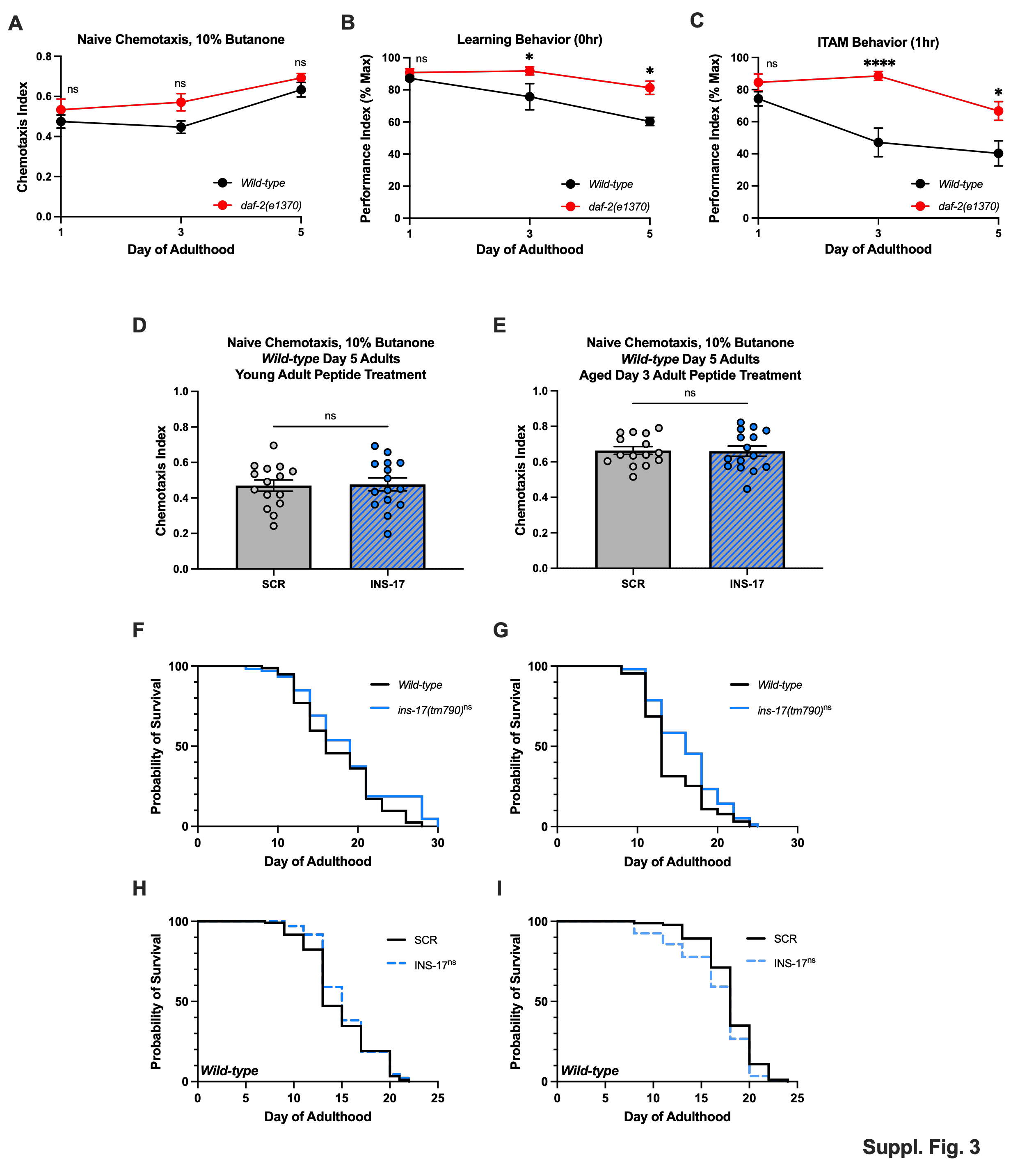

Supplement: S3 Fig — (A) Wild-type animals and daf-2(e1370) mutants have increased naïve chemotaxis to 10% butanone with age. Two-way ANOVA with Bonferroni’s multiple comparisons test. Interaction between factors, p = 0.6087; timepoint, p < 0.0001; genotype, p = 0.0119. Mean ± SEM. n = 10 per genotype. ns, not significant (p > 0.05). (B) daf-2(e1370) mutants maintain learning and (C) ITAM abilities better than wild-type animals with age across Days 1, 3 and 5 of adulthood. Two-way ANOVA with Bonferroni’s multiple comparisons test. For S3B, interaction between factors, p = 0.2450; timepoint, p = 0.0032; genotype, p = 0.0016. For S3C, interaction between factors, p = 0.0573; timepoint, p = 0.0020; genotype, p < 0.0001. Mean ± SEM. n = 10–15 per genotype. *p < 0.05, ****p < 0.0001; ns, not significant (p > 0.05). (D) Wild-type animals treated with SCR or INS-17 at either the L4 stage or at (E) Day 3 of adulthood had no detectable phenotypic effects in naïve chemotaxis to 10% butanone at Day 5 of adulthood. Mann-Whitney test comparing ranks. Mean ± SEM. n = 15 per genotype. ns, not significant (p > 0.05). (F-G) Replicates 2 and 3 of data represented in Fig 3G. Probability of survival analysis demonstrates there is no significant difference in lifespan comparing wild-type animals and ins-17(tm790) animals. Mean/median ± SEM. n ≥ 100 per replicate. ns, not significant (p > 0.05). (H-I) Replicates 2 and 3 of data represented in Fig 3H. Probability of survival analysis for wild-type animals treated with SCR peptide compared to wild-type animals fed INS-17 peptide shows no significant difference in average lifespan. Mean/median ± SEM. n ≥ 100 per replicate. ns, not significant (p > 0.05). (TIFF) [file pgen.1012130.s003.tiff]

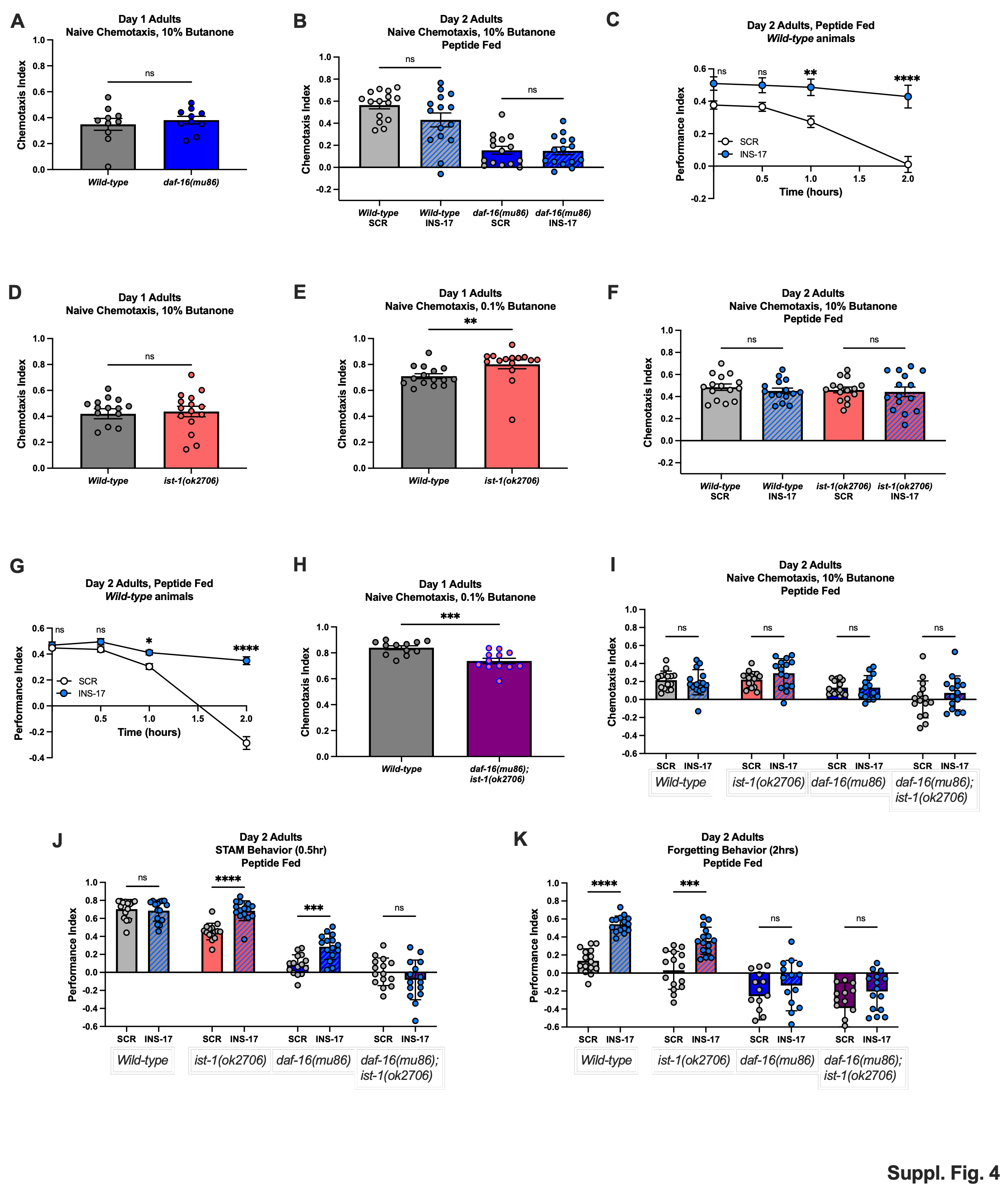

Supplement: S4 Fig — (A) Wild-type animals and daf-16(mu86) mutants have naïve chemotaxis behavior to 10% butanone that is not significantly different. Mann-Whitney test comparing ranks. Mean ± SEM. n = 10 per genotype. ns, not significant (p > 0.05). (B) SCR and INS-17 peptide treatments have no detectable effects on naïve chemotaxis behavior of wild-type worms nor daf-16 mutants to 10% butanone. One-way ANOVA with Bonferroni’s multiple comparisons tests (p < 0.0001). Mean ± SEM. n = 15 per genotype. ns, not significant (p > 0.05). (C) Confirmation for experiments in Fig 4B that INS-17 peptide feeding resulted in wild-type animals fed INS-17 to display memory behavior at two hours compared to animals fed SCR, which do not display memory behavior at two hours (forgetting). Two-way ANOVA with Bonferroni’s multiple comparisons test. Interaction between factors, p = 0.0058; timepoint, p < 0.0001; treatment, p < 0.0001. Mean ± SEM. n = 15 per genotype. **p < 0.01, ****p < 0.0001; ns, not significant (p > 0.05). (D) Wild-type animals and ist-1(ok2706) mutants have naïve chemotaxis behavior to 10% butanone that is not significantly different. Mann-Whitney test comparing ranks. Mean ± SEM. n = 15 per genotype. ns, not significant (p > 0.5). Additionally, (E) ist-1(ok2706) naïve chemotaxis to attractive 0.1% butanone, while significantly higher than wild-type, is not impaired. Mann-Whitney test comparing ranks. Mean ± SEM. n = 10 per genotype. **p < 0.01. (F) SCR and INS-17 peptide treatments have no detectable effects on naïve chemotaxis behavior of wild-type worms nor ist-1 mutants to 10% butanone. One-way ANOVA with Bonferroni’s multiple comparisons test (p > 0.05, ns). Mean ± SEM. n = 15 per genotype. ns, not significant (p > 0.05). (G) Confirmation for experiments in Fig 4D that INS-17 peptide feeding resulted in wild-type animals fed INS-17 to display memory behavior at two hours compared to animals fed SCR, which do not display memory behavior at two hours (forgetting). Two-way ANOVA wit [file pgen.1012130.s004.tiff]

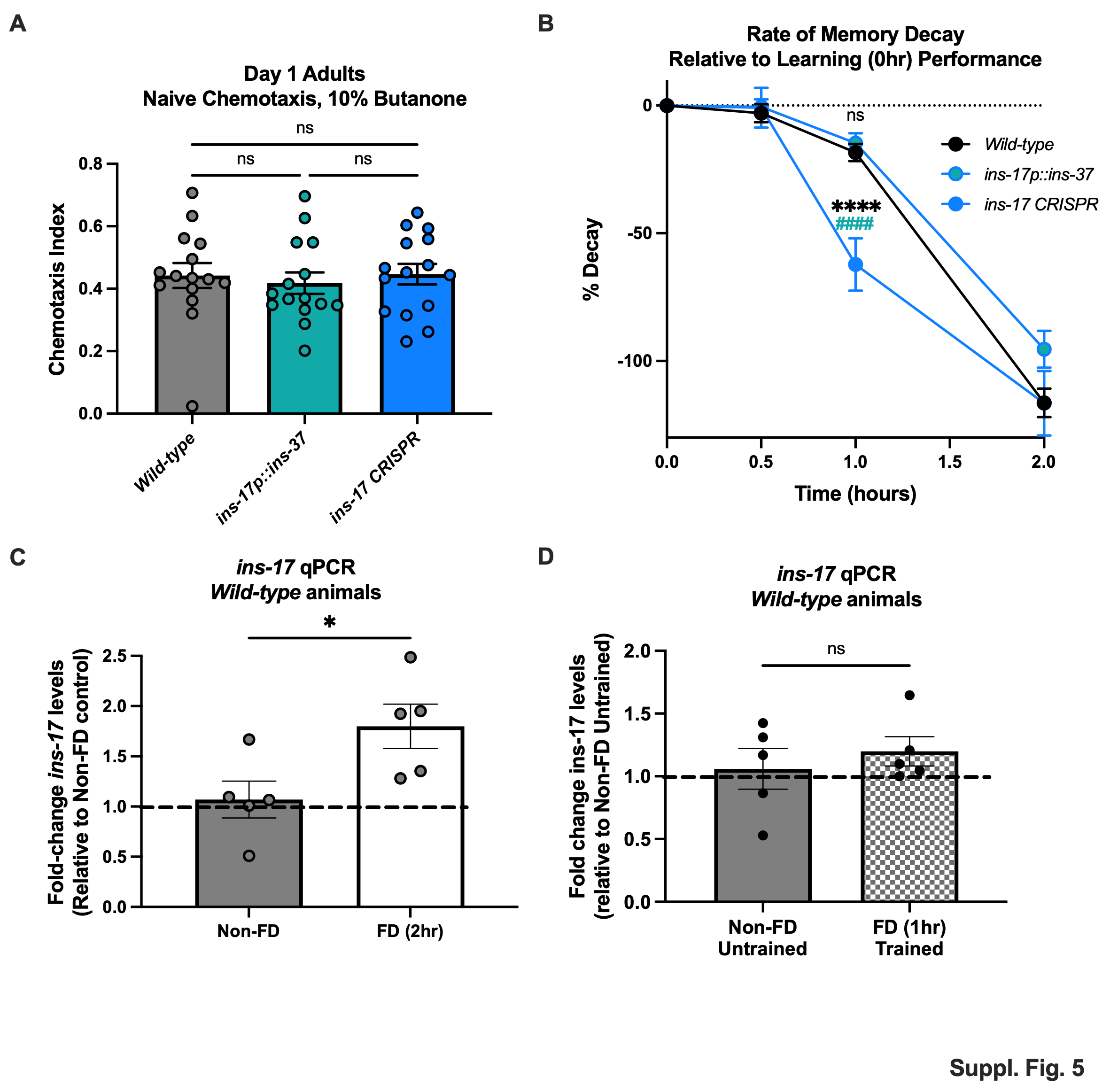

Supplement: S5 Fig — (A) There are no detectable differences in naïve chemotaxis to 10% butanone in wild-type animals, ins-17 CRISPR KO animals (ins-17(knu1320)), nor ins-17p::ins-37 (ins-17(knu1330[ins-37])) animals. One-way ANOVA with Bonferroni’s multiple comparisons test (p > 0.05, ns). Mean ± SEM. n = 15 per genotype. ns, not significant (p > 0.05). (B) CRISPR KO of ins-17 results in significant memory decay at the ITAM timepoint compared to wild-type and ins-17p::ins-37 behavioral decay at this timepoint. Two-way ANOVA with Bonferroni’s multiple comparisons test. Interaction between factors, p = 0.0002; timepoint, p < 0.0001; genotype, p = 0.0005. Mean ± SEM. n = 15 per genotype. * is performance compared to wild-type and # is performance relative to ins-17p::ins-37 performance. ****/####p < 0.0001. (C) qRT-PCR of ins-17 mRNA levels in Day 1 adult wild-type animals show that two hours of food-deprivation results in increased ins-17 transcript levels. The dotted line represents a fold-change of 1 relative to the average of non-FD control values, indicating no change between conditions at that level. Mann-Whitney test comparing ranks. Mean ± SEM. n = 5 per genotype. *p < 0.05. (D) Untrained wild-type animals have ins-17 transcript levels not significantly different from animals that underwent the FD step prior to training. The dotted line represents a fold-change of 1 relative to the average of untrained, non-FD control values, indicating no change between conditions at that level. Mann-Whitney test comparing ranks. Mean ± SEM. n = 5 per genotype. ns, not significant (p > 0.5). (TIFF) [file pgen.1012130.s005.tiff]

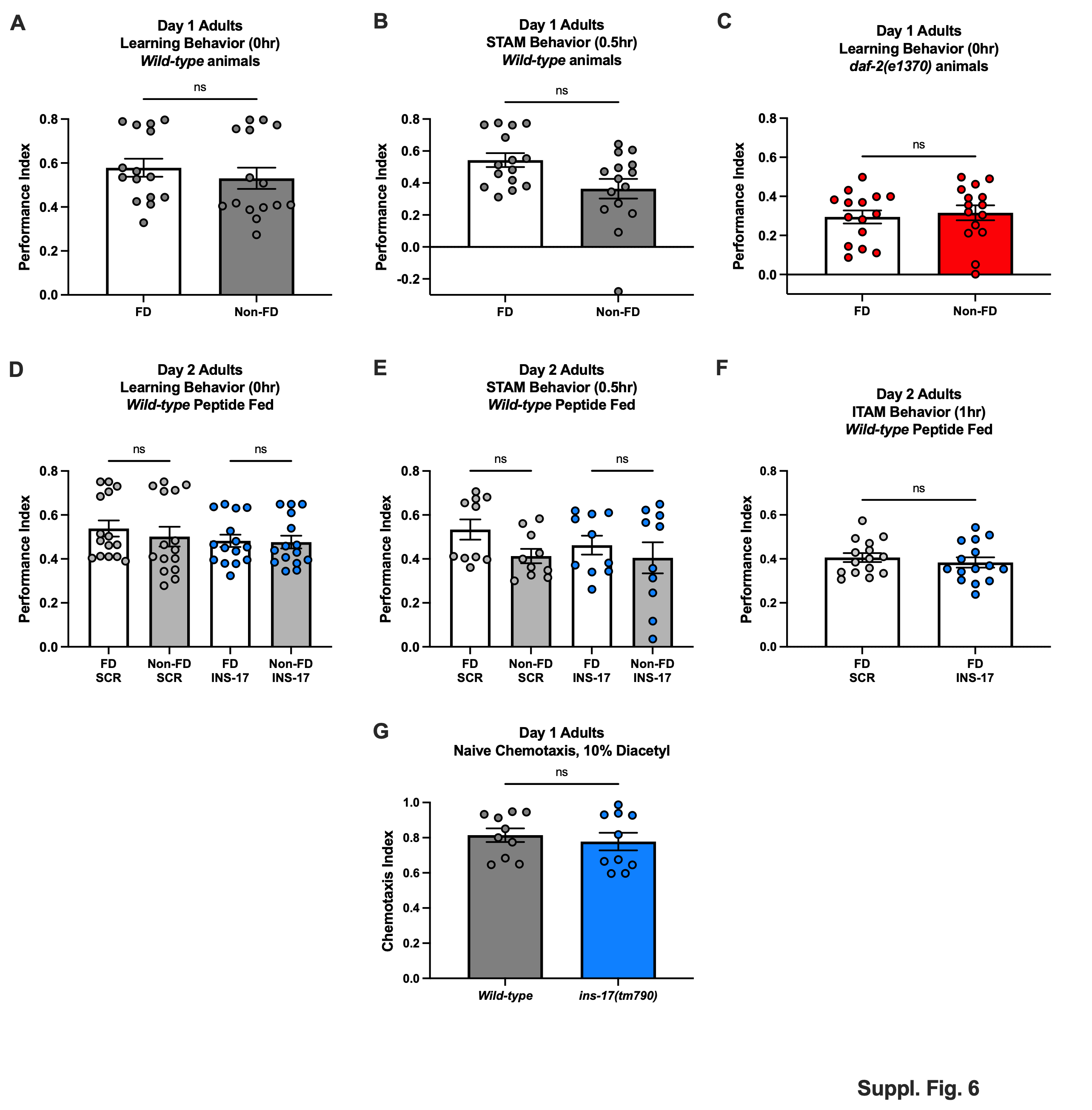

Supplement: S6 Fig — (A) Wild-type animals FD or non-FD prior to training showed no significant differences between learning and (B) STAM behaviors. Mann-Whitney test comparing ranks. Mean ± SEM. n = 15 per genotype. ns, not significant (p > 0.05). (C) Food-deprivation prior to training (1 CS-US pairing) does not enhance daf-2(e1370) learning performance. Mann-Whitney test comparing ranks. Mean ± SEM. n = 15 per genotype. ns, not significant (p > 0.05). (D) Wild-type animals treated with either SCR or INS-17 peptide had learning and (E) STAM behaviors not significantly different between the FD SCR or INS-17 fed groups and the non-FD SCR or INS-17 fed groups. One-way ANOVA with Bonferroni’s multiple comparisons test (p > 0.05, ns). Mean ± SEM. n = 10–15 per genotype. ns, not significant (p > 0.05). Meanwhile, (F) FD wild-type animals fed SCR had ITAM performance similar to INS-17 fed counterparts. Mann-Whitney test comparing ranks. Mean ± SEM. n = 10–15 per genotype. ns, not significant (p > 0.05). (G) ins-17(tm790) animals have no detectable deficits in chemotaxis to the highly attractive 1% diacetyl compared to wild-type animals. Mann-Whitney test comparing ranks. Mean ± SEM. n = 10 per genotype. ns, not significant (p > 0.05). (TIFF) [file pgen.1012130.s006.tiff]

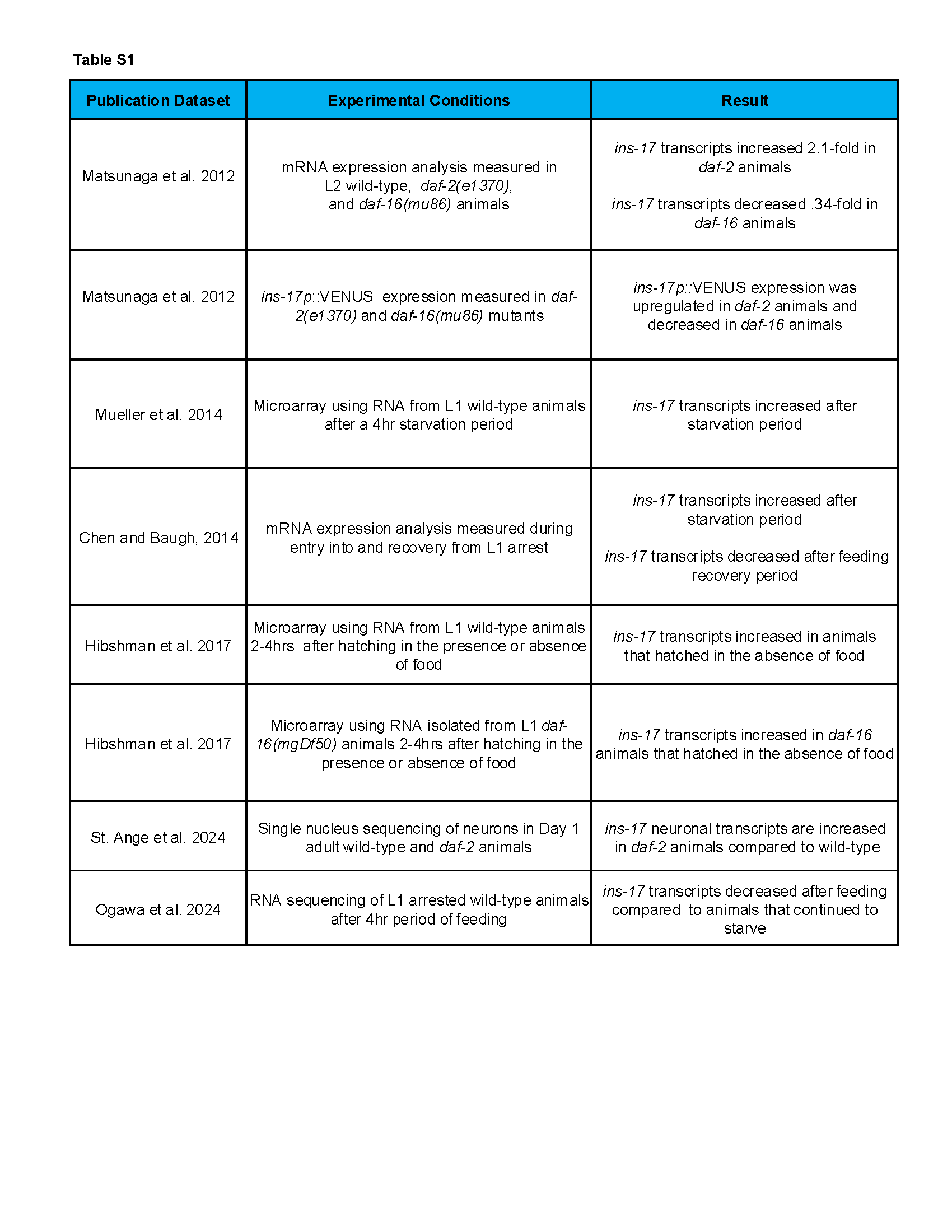

Supplement: S1 Table — (TIFF) [file pgen.1012130.s007.tiff]
